# Computational Exploration of the Ability of the 2‑Methyltetrols Produced from Photooxidation of Isoprene to Form Prenucleation Complexes

**DOI:** 10.1021/acsomega.5c01981

**Published:** 2025-06-06

**Authors:** Conor J. Bready, Alexandra E. Sorescu, Caroline S. Glick, George C. Shields

**Affiliations:** Department of Chemistry, 3628 Furman University, 3300 Poinsett Highway, Greenville, South Carolina 29613, United States

## Abstract

A central question in the formation of secondary aerosols is whether organic molecules participate in the formation of prenucleation clusters or are they only adsorbed after formation of larger aerosols? The difficulty in understanding the role of organic molecules in aerosol formation is that there are very few studies of prenucleation clusters produced from various organics and sulfuric acid, so it is uncertain whether organic compounds form prenucleation clusters. Isoprene is the most abundant volatile biogenic organic compound (VOC) emitted into the atmosphere, accounting for about 70% of biogenic VOC emissions, excluding methane. Each year, approximately 600 teragrams of isoprene enter the atmosphere, primarily from natural sources like vegetation. This makes it a significant component of atmospheric organic molecules, much more prevalent than other VOCs emitted by plants or anthropogenic activities. Photooxidation of isoprene produces the diastereomeric tetrols, 2-methylthreitol and 2-methylerythritol, which contain four hydroxyl groups. We completed a comprehensive conformational search of both tetrols, and extensively explored the potential energy surfaces of these tetrols complexed with sulfuric acid and water. We report the vast ensemble of structures that are within 1 kcal/mol of the DLPNO-CCSD­(T)/CBS//ωB97X-D/6-31++G** minimum for each system. These high level Δ*G*° values for each system were used to estimate the concentrations of all the possible complexes from these molecules in the lower troposphere. At the upper limit of tetrol concentration, we find that the two diastereomers will bind to one to three water molecules in high concentrations. However, formation of sulfuric acid–tetrol–water complexes lead to lower concentrations, leading us to suggest that these tetrols are unlikely to be involved in the formation of prenucleation clusters that will lead to further aerosol growth. Researchers should continue the search for organic molecules that lead to prenucleation.

## Graphical abstract

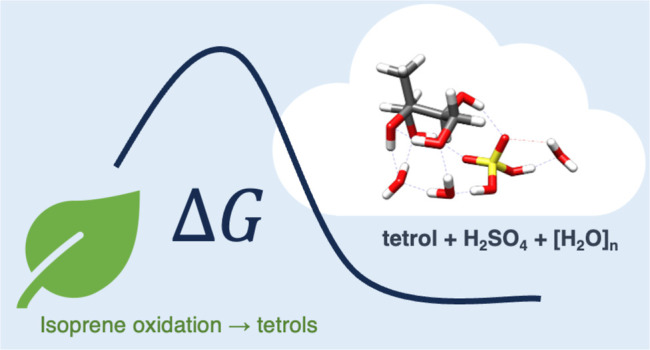

## Introduction

Aerosols in the atmosphere act as cloud condensation nuclei (CCN) and scatter and absorb solar light.^1–3^ The magnitude of the cooling effect produced by aerosols and clouds is the largest uncertainty in understanding how aerosols modulate climate change.^4^ Aerosols can enter the atmosphere directly from sea spray and desert dust or through emission from smoke caused by factories and forest fires. In addition, secondary aerosols develop from gas vapors in a process known as new particle formation (NPF). In NPF, the first step is the formation of prenucleation clusters, which can grow until they reach critical cluster size. A central question in the formation of secondary aerosols is the role of organic molecules: do organics participate in the formation of prenucleation clusters or are they only adsorbed after initial aerosol formation?^3^ Although numerous studies have examined prenucleation clusters involving various organics and sulfuric acid, the understanding of organic contributions to cluster formation remains limited.^3^ Formic acid has been studied using computational chemistry, and it forms a prenucleation cluster with sulfuric acid that is as strong and stable as the formation of an ammonia-sulfuric acid-nucleation cluster, a consequence of the strong hydrogen bonding present in the geometry of the sulfuric acid/formic acid dimer.^5,6^ Yet, cluster population dynamics simulations predict that formic acid has a miniscule effect on the formation of methanesulfonic acid clusters that include ammonia and amine bases.^7^ The same is true for sulfuric acid and amine bases, with the only significant prediction being that the sulfuric acid/dimethylamine system is enhanced by 21% relative to the same system without formic acid present.^7^ This is in line with previous work that shows despite the high concentration of formic acid in the atmosphere,^8^ it appears to stabilize sulfuric acid/water clusters without leading to further cluster growth.^9^

Figure 1 outlines the general scheme of what we know about the formation or various organic compounds in the atmosphere.^3^ Volatile organic compounds (VOCs) are molecules directly emitted into the gas phase from their various sources. Once these compounds enter the atmosphere, they react with atmospheric oxidants, such as hydroxyl radicals and ozone.^3^ Reactions with oxygen form oxidized species with different functional groups that are capable of forming hydrogen-bonded clusters with sulfuric acid and water. If organics are part of an aerosol formation process, they are called secondary organic aerosols, or SOAs. Isoprene, which is emitted by plants especially during hot weather, is one of the major organic compounds in the atmosphere, making up approximately 50% of the total VOCs emitted by plants.^10,11^ Isoprene has a short lifetime in the atmosphere, so it does not form SOAs. Instead, photooxidation of isoprene with oxygen changes its molecular structure. Hydroxyl radicals, which are ubiquitous in the atmosphere, oxidize isoprene. The initial photooxidation reactions of the hydroxyl radical with isoprene create two diastereomeric 2-methyltetrols: 2-methylthreitol and 2-methylerythritol. The 2-methyltetrols have the isoprene skeleton and four hydroxyl groups suitable for formation of hydrogen bonds, and therefore are candidates for organic molecules that could lead to SOA formation.^12–16^ We note that 2-methyltetrols are primarily condensed-phase products and are not abundant in the gas phase. To date, there have been no field campaigns that have identified the 2-methyltetrols in the atmosphere. Nevertheless, their highly oxygenated structure, with four hydroxyl groups, makes them valuable model compounds for probing cluster formation.

**Figure 1:**
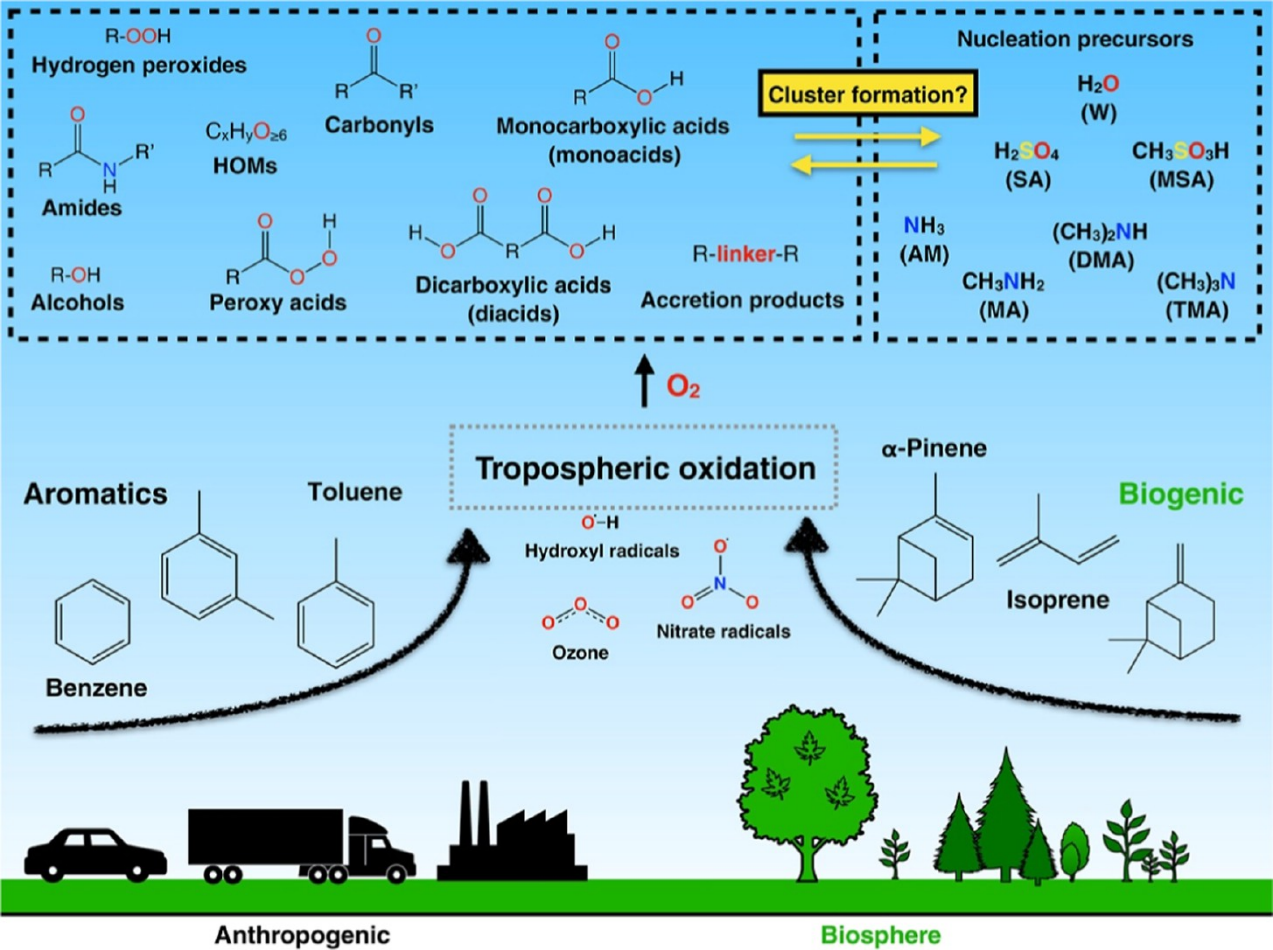
Diversity of organics present in the atmosphere. Reprinted with permission from ref 3. Copyright 2023 Wiley Periodicals, Inc.

In this paper we explore the hypothesis that the 2-methyltetrols could form prenucleation complexes with sulfuric acid. We determine the lowest energy structures and thermodynamic trends of 2-methylthreitol and 2-methylerythritol involving (H_2_O)_*n*_ (*n* = 0–4) as well as those containing H_2_SO_4_ and (H_2_O)_*n*_ (*n* = 0–3). From the Gibbs free energies of all of these structures and estimates of the starting concentrations of 2-methylthreitol, 2-methylerythritol, sulfuric acid, and water we estimate the concentrations of all possible combinations of these molecules. These isoprene-derived compounds are important to study since isoprene plays a significant role in nature and the prenucleation ability of the 2-methyltetrols is unknown.

## Methods

Figure 2 outlines the following steps of the funnel methodology that has been extensively used to generate the lowest-energy structures.^3,17,18^

**Figure 2:**
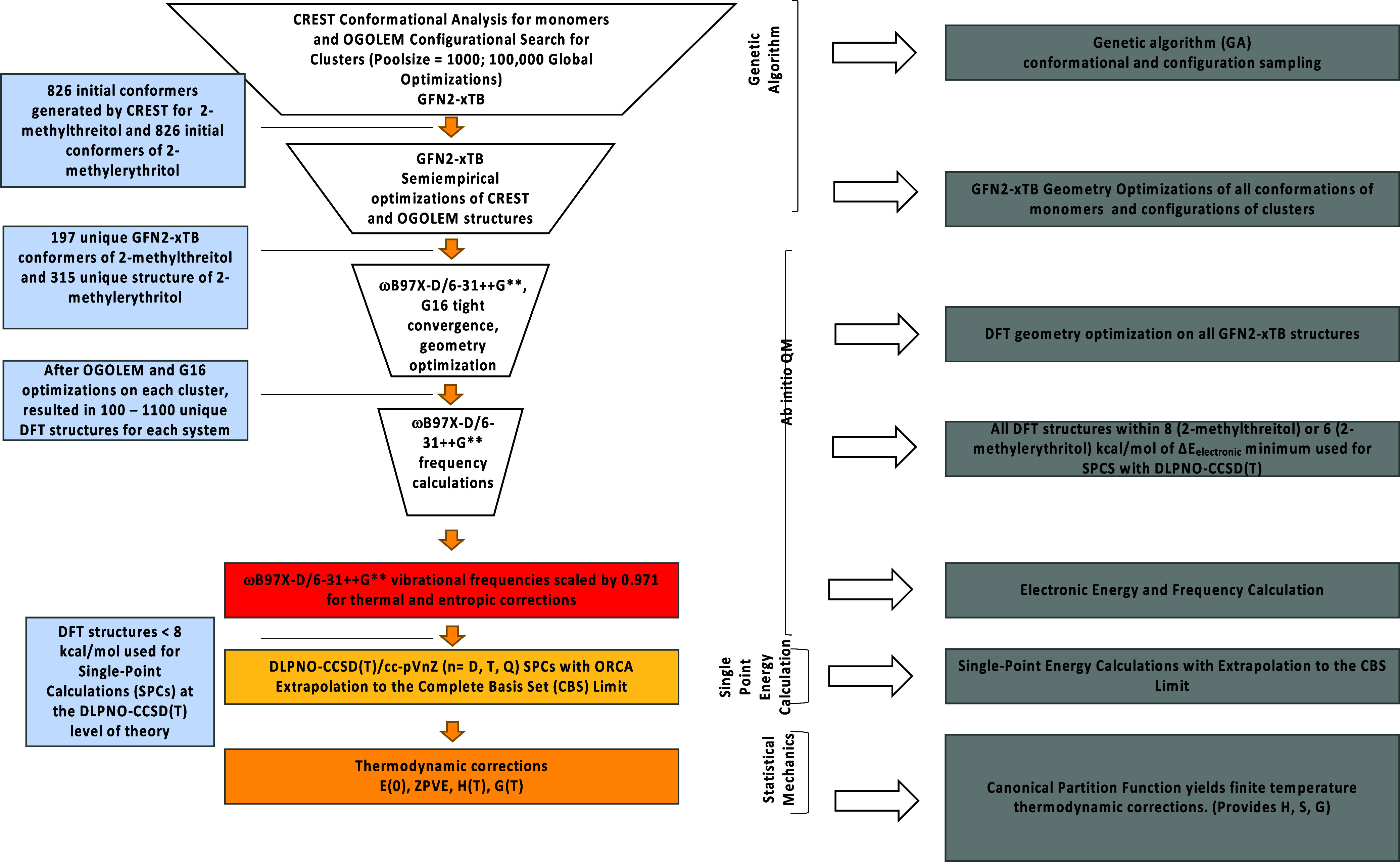
Funnel methodology applied in generating molecular structures and determining the relative and absolute Gibbs free energies for each system.

In the first two steps, CREST (Conformer-Rotamer-Ensemble Sampling Tool)^19^ was applied as the conformational sampling method with the semiempirical GFN2-xTB method^20,21^ to generate 197 GFN2 conformers of 2-methylthreitol and 315 GFN2 conformers of 2-methylerythritol. In the third step density functional theory (DFT) was used to optimize all of the GFN2 conformers produced from the CREST routine, and to calculate the vibrational frequencies for each conformer (step four). The ωB97X functional^22,23^ was employed in the Gaussian16 Rev. B01 program^24^ with the 6-31++G** basis set^25–28^ and Grimme’s dispersion correction^29^ (i.e., ωB97X-D) to optimize all of the initial conformations and calculate vibrational frequencies. Tight convergence criteria was set for both the self-consistent field method and geometry optimization, and duplicate structures were removed from the collection,^30^ resulting in 156 DFT structures of 2-methylthreitol and 223 DFT structures of 2-methylerythritol. These structures were then used for single point energy calculations with the DLPNO-CCSD­(T)^31–34^ coupled cluster electronic theory with the ORCA program^35^ to obtain high level electronic energies with the correlation consistent Dunning basis sets, cc-pVnZ (n = D, T, Q),^36,37^ which were then extrapolated to the complete basis set (CBS) limit.^38^ The DFT frequencies were scaled by 0.971 ^39^ and used to determine the thermodynamic corrections *H*°, *S*°, and *G*° at 216.65, 273.15, and 298.15 K and 1 atm pressure.^40,41^ We note that the low-frequency modes were retained in the thermochemical analysis and scaled by 0.971. While this is a commonly used approximation, it may lead to errors in entropy contributions, affecting the computed Gibbs free energies.^42^ The thermodynamic corrections were then combined with the DLPNO-CCSD­(T) electronic energies in the final step to obtain the Gibbs free energies for all clusters. We then used a cutoff of one kcal/mol for the Gibbs free energies at 298.15 K to produce the initial set of 2-methyltetrols which were used for the simulations with water and sulfuric acid, resulting in three initial 2-methylthreitol conformers and 15 initial 2-methylerythritol conformations of the 2-methyltetrol monomers. Using the cutoff of one kcal/mol is computationally efficient, but it may limit the structural diversity explored during cluster sampling. Future studies may consider including select higher-energy conformers, which may contribute nontrivially to cluster formation.

For the simulations of each 2-methyltetrol conformer with water and sulfuric acid, we used the OGOLEM evolutionary algorithm,^43^ which is a global cluster structure optimization program for mixtures of flexible molecules, to produce a final set of semiempirical structures optimized with the GFN2-xTB method.^20,21^ Each 2-methyltetrol conformer was combined with one, two, three, or four waters to examine the hydrogen bonding ability of each isomer with a handful of waters. Subsequently each 2-methyltetrol conformer was combined with a sulfuric acid molecule and zero, one, two, or three waters. A pool size of 1000 configurations was used for each simulation and the results from three OGOLEM runs for each system using the three conformers of 2-methylthreitol were combined. Similarly, the results from the 15 OGOLEM runs for each system using the 15 conformers of 2-methylerythritol were combined for each 2-methyltetrol system. Thus, a total of 144 OGOLEM simulations were run, 24 for 2-methylthreitol and 120 for 2-methylerythritol. Unique structures were determined by examining the GFN2 rotational constants and relative energies—structures with rotational constants within 1% and relative energies <0.00015 au (∼0.1 kcal/mol) were considered identical. A final, deduplicated set of 2-methylthreitol and 2-methylerythritol isomers for every system was then geometry optimized in the next step using DFT. The ωB97X-D functional^22,23^ in Gaussian16 ^24^ with the 6-31++G** basis set^25–28^ was utilized to calculate minimum energy structures and the enthalpic and entropic values^40,41^ in order to obtain Gibbs free energies. Then, DLPNO-CCSD­(T)^31–34^ calculations were run with ORCA^35^ to obtain high electronic-level energies with the basis sets, cc-pVnZ (n = D, T, Q),^36,37^ which were then extrapolated to the complete basis set (CBS) limit.^38^ A cutoff value of eight kcal/mol on the ωB97X-D/6-31++G** electronic energies was used to ensure that high entropy structures on the DLPNO-CCSD­(T) potential energy surface (PES) were not missed for the 2-methylthreitol system. After examining the output this cutoff was lowered to six kcal/mol for the 2-methylerythritol, which we determined was reasonable given the results from 2-methylthreitol and the larger number of simulations (120) that were run for this isomeric system. Finally, the DLPNO-CCSD­(T)/CBS electronic energies were combined with the ωB97X-D statistical mechanical thermodynamic values to obtain *S*°, *H*°, and *G*° for each cluster at 217 and 298 K.^40,41^ Estimated equilibrium concentrations of the 2-methyltetrol isomers complexed with water and sulfuric acid at 298.15 K were calculated from the stepwise Δ*G*_298.15_ values for formation of each possible cluster and estimated initial concentrations of the two 2-methyltetrol monomers, water, and sulfuric acid.^12,41,44–46^

We present Δ*G*_298.15_ of formation, and corresponding concentrations, using two approaches. In one approach, we calculate Δ*G*_298.15_ of formation using the Gibbs free energies of the minimum energy structures. In another approach, we calculate Δ*G*_298.15_ of formation using a corrected Gibbs free energy of the minimum structures, where we add the free energy contribution from other conformers.^47^ This correction is computed as

$$G=-RT\;\ln\left(\sum_{k}e^{-\Delta G_{k}/RT}\right)$$

where Δ*G*_*k*_ is the Gibbs free energy of conformer *k* relative to the lowest. The calculated contributions are listed in the Supporting Information.

## Results and Discussion

### Structures with the Lowest Relative Gibbs Free Energies

#### Monomers

All of the figures contain all structures that are within one kcal/mol of the DLPNO-CCSD­(T)/CBS//ωB97X-D/6-31++G** electronic energy minimum (Δ*E*_el_) or either of the 217 or 298 K Gibbs free energy minima. In every figure, hydrogen bonds, which we defined as having O–H···O bond angles between 140 and 180° and an H···O distance of less than 2.2 Å, are depicted by blue lines. Close contacts less than 140° or H···O distances greater than 2.2 Å are defined as van der Waals contacts and depicted by red lines. Each figure contains the DLPNO-CCSD­(T)/CBS//ωB97X-D/6-31++G** relative electronic and Gibbs free energies for the structures. In the descriptions in this section, we will refer to the relative Gibbs free energies at 298.15 K, Δ*G*_298.15_, when describing the structures in the figures. Figure 3 contains the three initial conformations of 2-methylthreitol used in the OGOLEM simulations.

**Figure 3:**
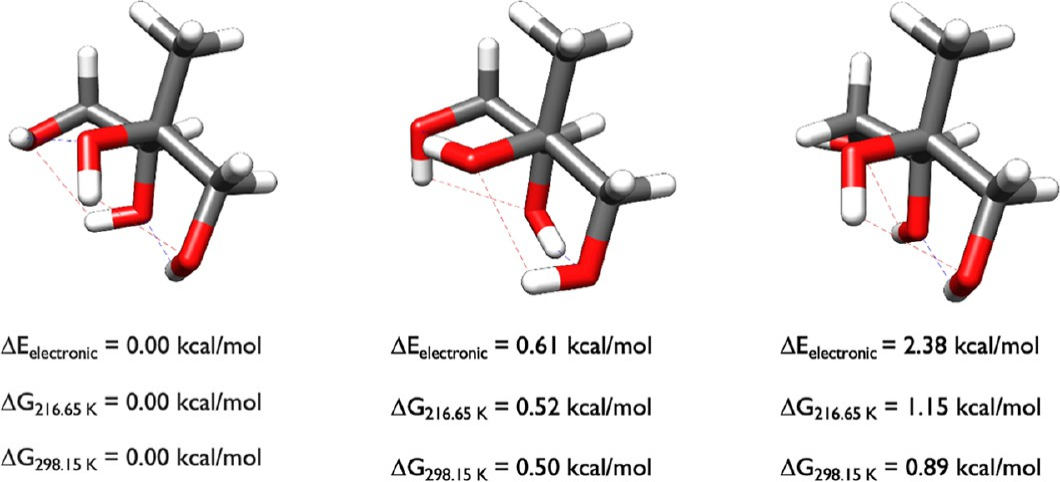
Starting structures of 2-methylthreitol calculated at the DLPNO-CCSD­(T)/cc-pVnZ//ωB97X-D/6-31++G** (*n* = D, T, Q) level of theory, where the energies have been extrapolated to the CBS limit.

The stereochemistry of 2-methylthreitol favors conformations where all of the hydroxyl groups interact with each other, forming intramolecular hydrogen bonds or van der Waals contacts, depending on the arbitrary nature of how we define an intramolecular hydrogen bond. Intramolecular hydrogen bonds can change the cis/trans electronic energy preference of diastereomeric 1,2-dialkyl-2,3-epoxycyclopentanol diastereomers and their thiiriane, aziridine and phosphirane analogues by up to 3–4 kcal/mol.^48,49^ Intramolecular hydrogen bonds are more difficult to define by geometry alone, and here we reduce the hydrogen bond angle criteria to 130° in our description. The first two structures have two intramolecular hydrogen bonds (1.98 Å, 133° and 2.02 Å, 134°; 1.94 Å, 144.5° and 1.88 Å, 146°), as indicated by the blue dashed lines, and two van der Waals interactions indicated by the red dashed lines (2.11 Å, 119° and 2.08 Å, 120°; 2.32 Å, 105°; 2.22 Å, 108°) whereas the third one, which is over 2 kcal/mol higher in electronic energy, is higher in entropy and is probably best described as having one intramolecular hydrogen bond (1.96 Å, 131°) and three van der Waals interactions (2.12 Å, 118° and 2.12 Å, 114° and 2.49 Å, 130°). The Gibbs free energy of the third isomer is within 0.9 kcal/mol of the free energy minimum, illustrating the impact of higher entropy for the third isomer. It also illustrates why using a high cutoff (6 kcal/mol) for the DFT energies is important to ensure that CCSD­(T) calculations were performed on all potential minima.

The starting structures of 2-methylerythritol in Figure 4 are much more diverse than the 2-methylthreitol conformers in Figure 3, which is a function of the stereochemistry. The switch in orientation about the Carbon stereocenter with the most unique functional groups (consisting of OH, CH_2_OH, CH_3_, and CH­(OH)­CH_2_OH) positions the hydroxyls differently between the 2-methyltetrol diastereomers, and this allows the 2-methylerythritol isomer to have many more interactions with the four hydroxyl groups in many different conformations. As seen in Figure 4, the conformations with a Δ*G*_298.15_ of 0.00, 0.49, 0.52, and 0.87 kcal/mol are similar, and contain one intramolecular hydrogen bond and two van der Waals interactions, with only the OH groups rotated differently. The structures with Δ*G*_298.15_ of 0.75 and 0.89 kcal/mol have similar shape; the structure at 0.75 kcal/mol has two van der Waals interactions while the one at 0.89 kcal/mol has one intramolecular hydrogen bond and a van der Waals interaction. The conformation at 0.80 kcal/mol contains three van der Waals interactions and has a similar orientation as the conformation at 0.98 kcal/mol.

**Figure 4:**
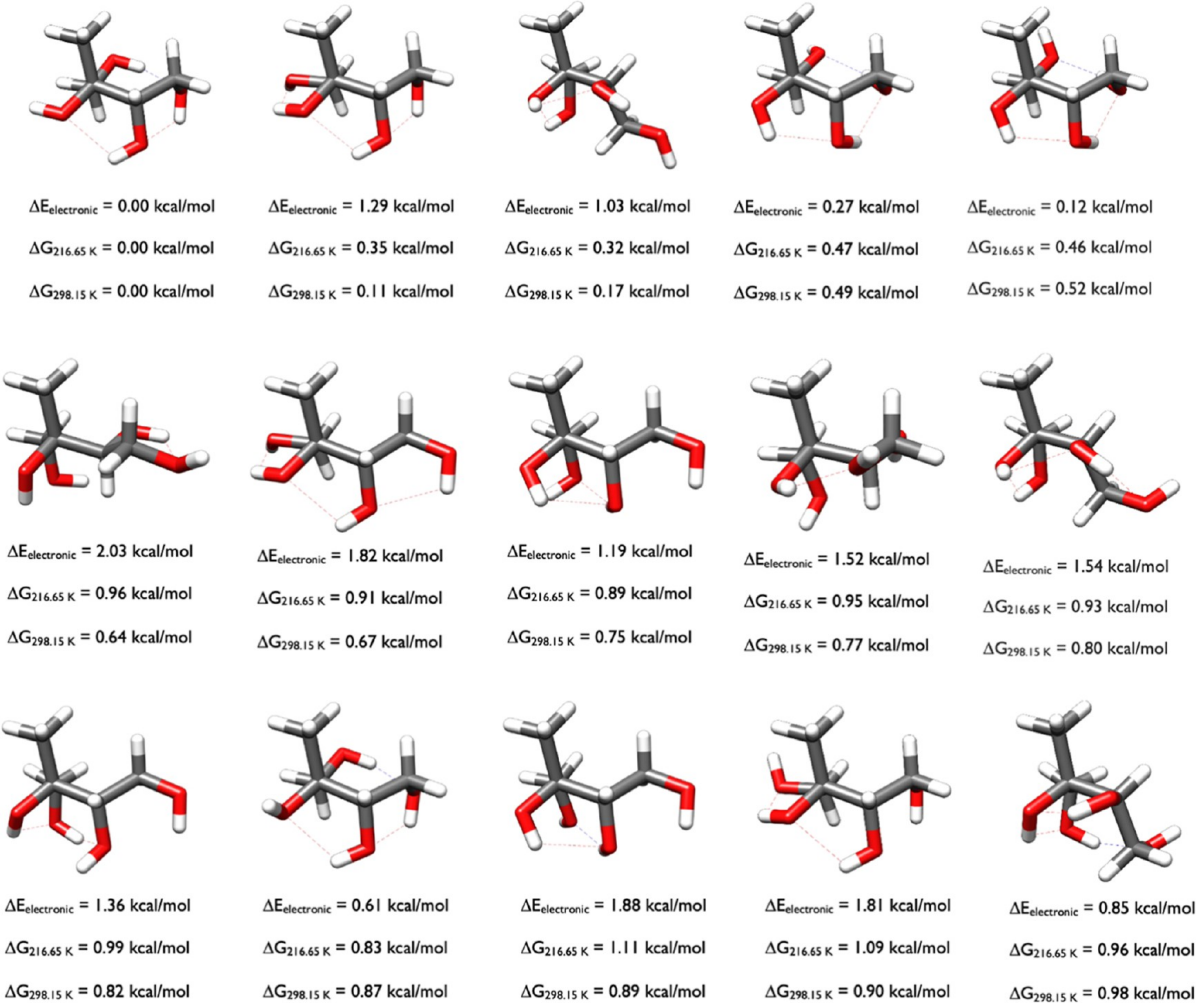
Starting structures of 2-methylerythritol calculated at the DLPNO-CCSD­(T)/cc-pVnZ//ωB97X-D/6-31++G** (*n* = D, T, Q) level of theory, where the energies have been extrapolated to the CBS limit.

#### 2-Methyltetrols with Water

Adding a water to 2-methylthreitol, Figure 5, shows that the first two structures of 2-methylthreitol have a similar shape and one van der Waals interaction. However, the lowest-energy Δ*G*_298.15_ cluster forms four hydrogen bonds, two intermolecular between the water and 2 OH groups and two intramolecular within 2-methylthreitol. The second lowest Gibbs free energy structure at 0.13 kcal/mol has three hydrogen bonds, two of which are intramolecular. The 2-methylthreitol molecules are congruent in these two isomers, and only the rotation of the water separates their structures and energies. The conformation at 0.34 kcal/mol has water bridging two hydroxyl groups forming two hydrogen bonds, two intramolecular hydrogen bonds between hydroxyl groups, and a van der Waals interaction between two hydroxyl groups of the cluster. Subtle interactions change the energies, as in the two clusters at 0.36 and 0.85 kcal/mol which have identical 2-methylthreitol geometries, with the water molecule interacting in different places.

**Figure 5:**
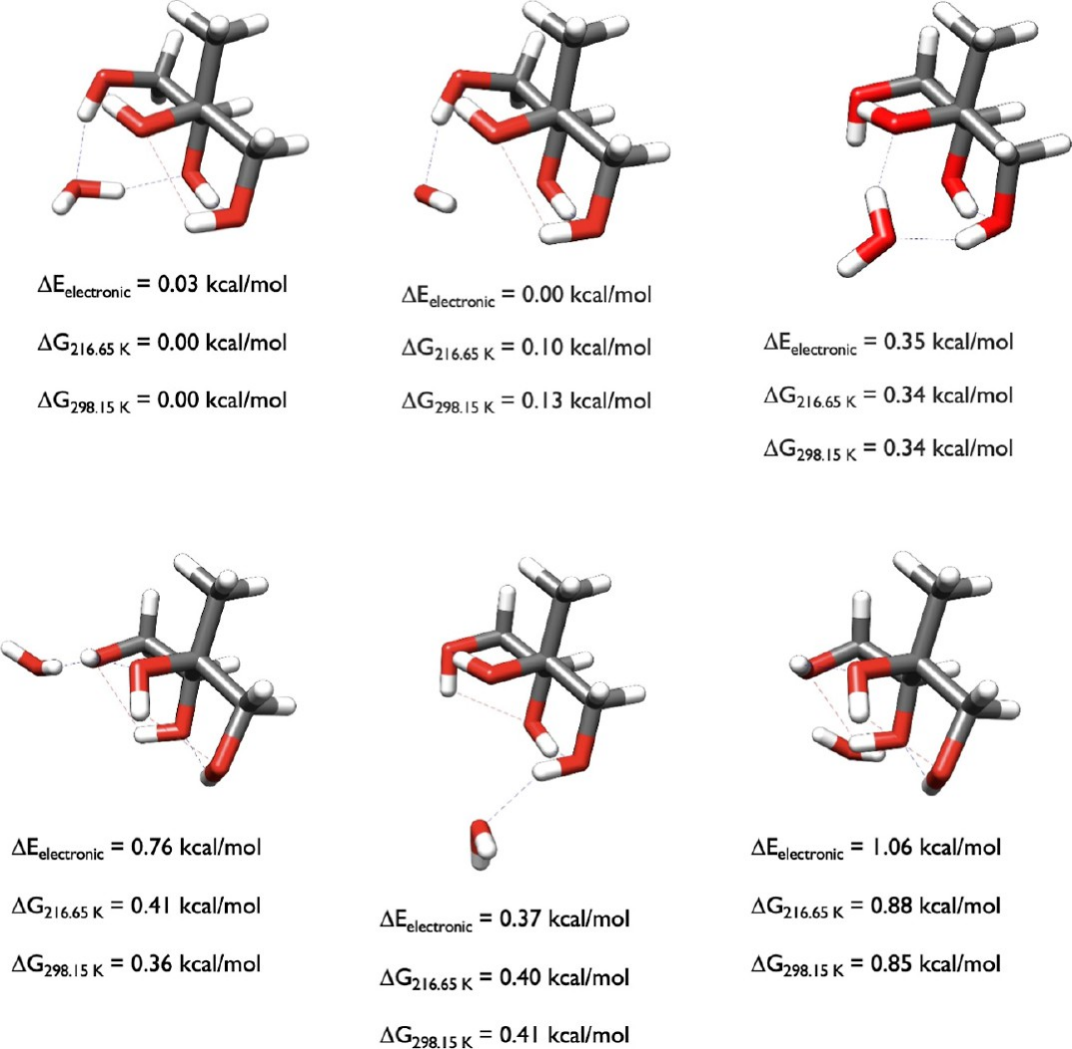
Lowest energy structures of 2-methylthreitol-H_2_O calculated at the DLPNO-CCSD­(T)/cc-pVnZ//ωB97X-D/6-31++G** (*n* = D, T, Q) level of theory, where the energies have been extrapolated to the CBS limit.

Adding two waters to 2-methylthreitol, Figure 6, results in 11 configurations. The 2-methylthreitol structures with Δ*G*_298.15_ of 0.00, 0.49, and 0.65 kcal/mol are congruent, and differ by the way the two waters are rotated in varied directions. A second motif applies to the clusters at 0.13, 0.51, 0.76, and 0.84 kcal/mol and a third motif is for the clusters with Δ*G*_298.15_ of 0.67, 0.73, and 0.85 kcal/mol. Most of the waters in these later structures are hydrogen bonded to each other.

**Figure 6:**
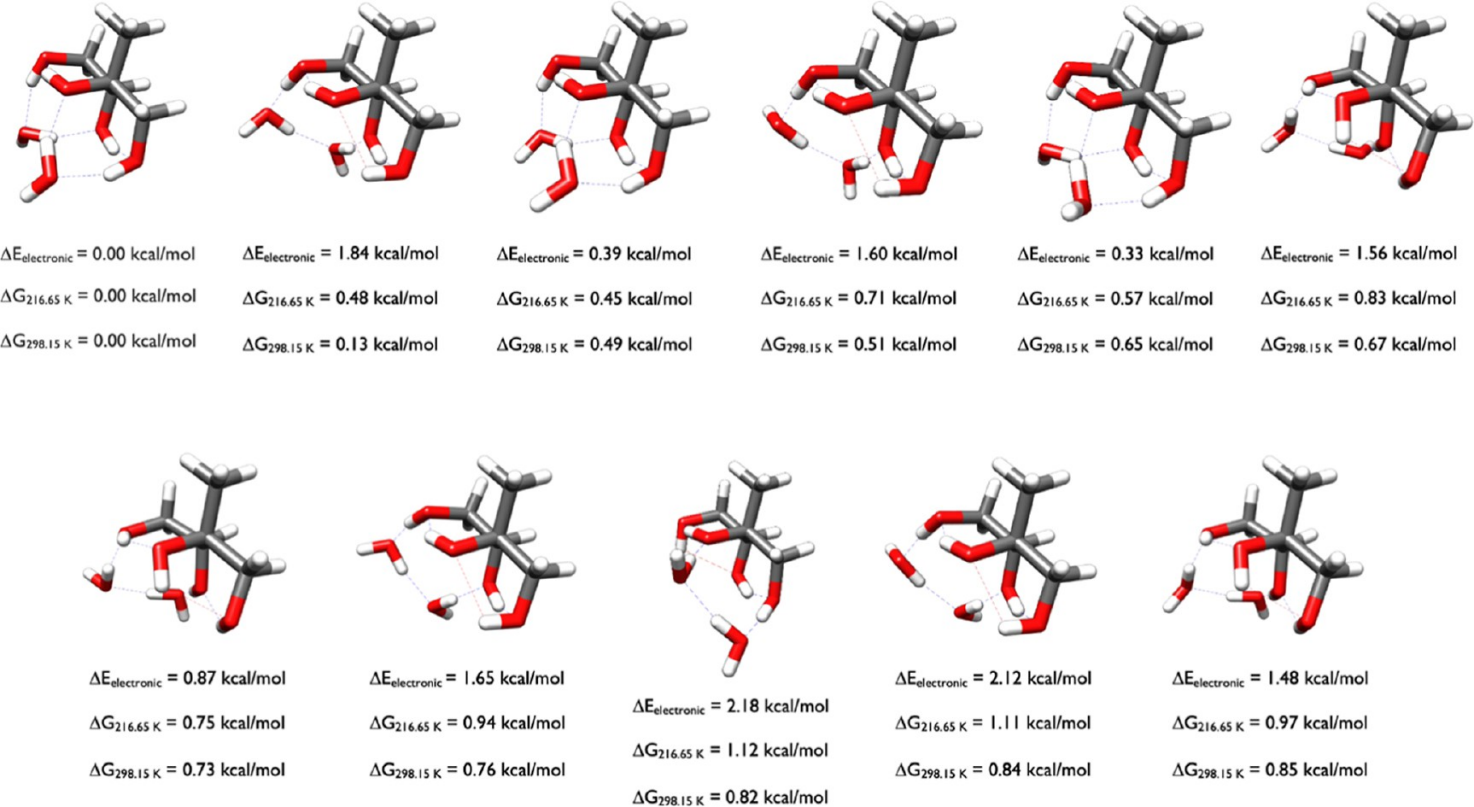
Lowest energy structures of 2-methylthreitol-(H_2_O)_2_ calculated at the DLPNO-CCSD­(T)/cc-pVnZ//ωB97X-D/6-31++G** (*n* = D, T, Q) level of theory, where the energies have been extrapolated to the CBS limit.

The PES with 2-methylthreitol and three waters is vast. In the OGOLEM search 1319 unique GFN2-xTBb configurations were produced, which when optimized with ωB97X-D collapsed to 779 unique DFT structures. Of those 779, 535 had ωB97X-D Δ*E*_el_ values within 8 kcal/mol of the minimum (normally we used a 6 kcal/mol cutoff but a larger cutoff was used for this system), and DLPNO-CCSD­(T)/CBS calculations on these 535 produced the energies in Figure 7. We note that the second structure, which has a DLPNO-CCSD­(T)/CBS Δ*E*_el_ value of 3.64 kcal/mol, was the 225th structure on the ωB97X-D PES (Δ*E*_el_ of 5.93 kcal/mol; Δ*G*_298.15_ of 1.96 kcal/mol). Figure 7 reveals that once three waters are added to 2-methylthreitol, all of the minima have the OH groups rotated to the same side of 2-methylthreitol, which allows for interactions between the hydroxyl groups and the waters, and the waters with each other. The intermolecular hydrogen bonds formed by waters with each other and the 2-methylthreitol hydroxyls are stronger than the intramolecular hydrogen bonds and van der Waals interactions between the 2-methylthreitol OH groups themselves. The 2-methylthreitol structures within each cluster are similar to the three initial monomer conformers in Figure 3. Thus, we learn that while adding one or two waters to 2-methylthreitol leads to a variety of different geometries, once three waters are added the initial starting conformations of the 2-methylthreitol monomer (Figure 3), these configurations often remain the most stable.

**Figure 7:**
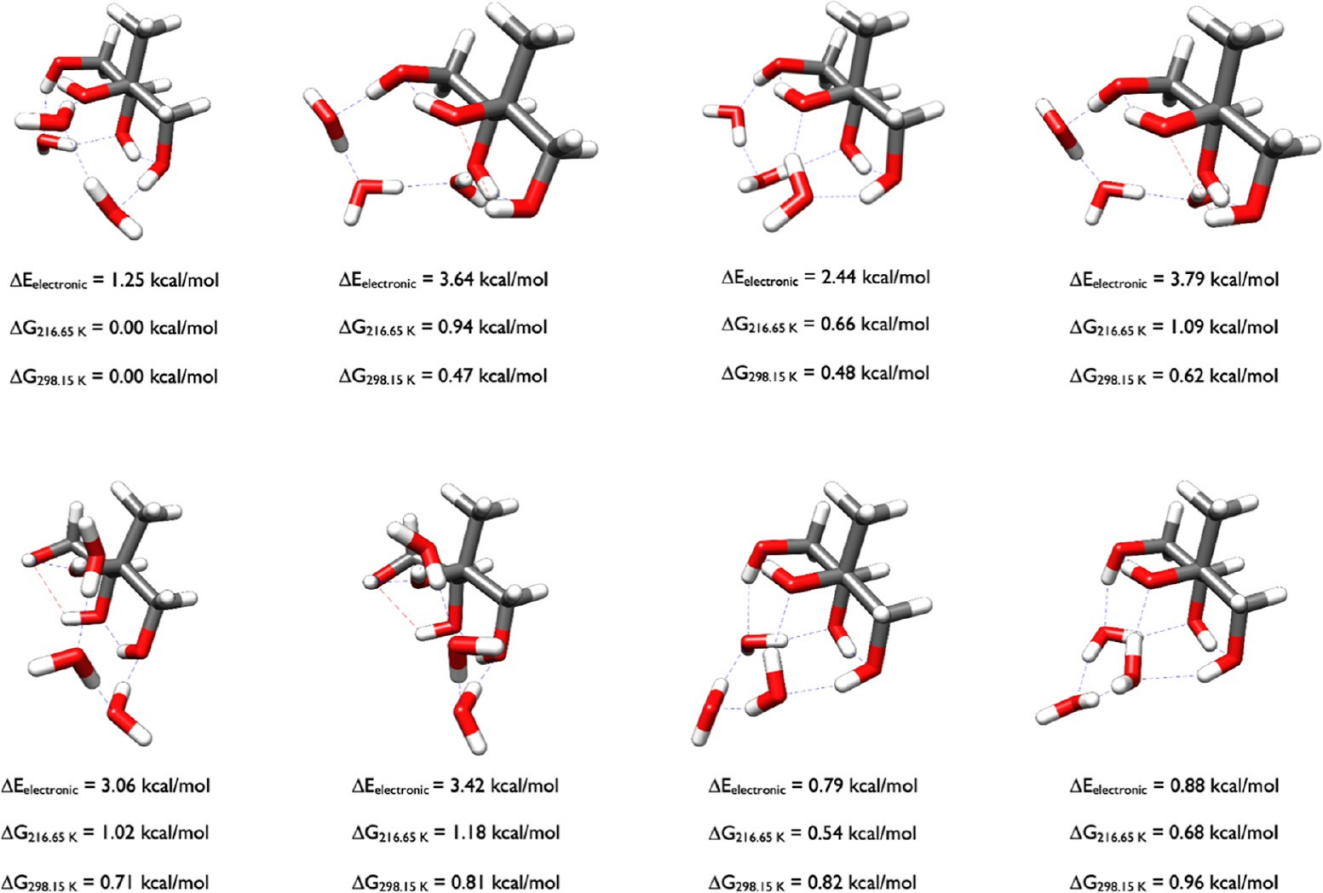
Lowest energy structures of 2-methylthreitol-(H_2_O)_3_ calculated at the DLPNO-CCSD­(T)/cc-pVnZ//ωB97X-D/6-31++G** (*n* = D, T, Q) level of theory, where the energies have been extrapolated to the CBS limit.

The 2-methylthreitol-(H_2_O)_4_ structures displayed in Figure 8 with Δ*G*_298.15_ values of 0.00, 0.35, 0.58, and 0.71 kcal/mol reveal that 2-methylthreitol geometries are congruent, and identical to the second conformer (0.50 kcal/mol) in Figure 3. An interesting feature is the presence of the water tetramer, which has been shown to be quite stable,^50,51^ and forms stabilizing interactions for the clusters with Δ*G*_298.15_ values of 0.36, 0.63, and 0.91 kcal/mol. As another comparison, the configurations at 0.06 and 0.24 kcal/mol are quite similar, with just a slight rotation of one of the water molecules giving rise to the difference in energies.

**Figure 8:**
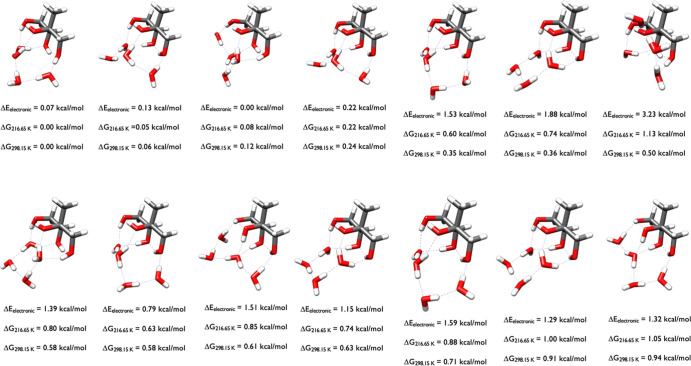
Lowest energy structures of 2-methylthreitol-(H_2_O)_4_ calculated at the DLPNO-CCSD­(T)/cc-pVnZ//ωB97X-D/6-31++G** (*n* = D, T, Q) level of theory, where the energies have been extrapolated to the CBS limit.

Figure 9 displays the ten 2-methylerythritol-H_2_O structures within 1 kcal/mol of the Gibbs free energy minimum. The OGOLEM simulations started from the 15 different conformers of 2-methylerythritol (Figure 4) and the addition of one water. The structures at 0.71 and 0.91 kcal/mol are most similar to the minimum energy structure in Figure 4, while the 0.99 kcal/mol structure is most similar to the 0.52 kcal/mol structure in Figure 4. The second structure in Figure 9, 0.19 kcal/mol above the minimum, has a 2-methylerythritol geometry closest to the 0.77 kcal/mol geometry in Figure 4.

**Figure 9:**
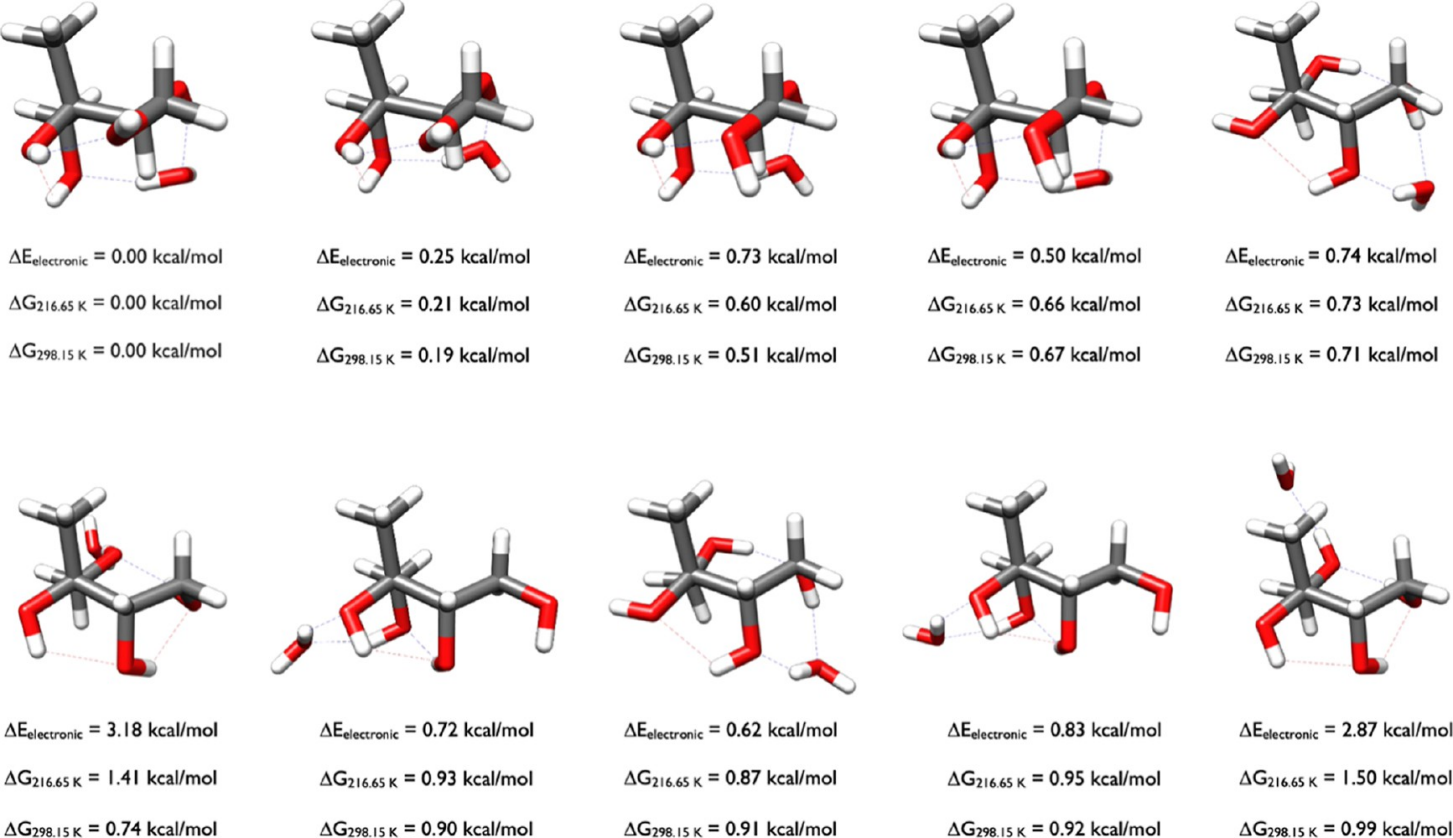
Lowest energy structures of 2-methylerythritol-H_2_O calculated at the DLPNO-CCSD­(T)/cc-pVnZ//ωB97X-D/6-31++G** (*n* = D, T, Q) level of theory, where the energies have been extrapolated to the CBS limit.

The water dimer is prevalent in all but one of the lowest energy configurations (0.89 kcal/mol) of the 2-methylerythritol-(H_2_O)_2_ system, forming a tetrameric structure with two hydroxyl groups (Figure 10). The water dimers bind to two different hydroxyl groups, forming a pseudotetramer to stabilize these clusters. The three lowest energy configurations for the 2-methylerythritol-(H_2_O)_2_ cluster are the first three structures depicted in Figure 10, with the same 2-methylerythritol geometry and slight variations in the water positions. The 2-methylerythritol geometries at 0.17, 0.21, and 0.22 kcal/mol retain congruence, again with slightly different water interactions. Another motif is represented by the structures with Δ*G*_298.15_ of 0.29 and 0.57 kcal/mol. A slightly different motif is represented by the two isomers at 0.74 and 0.78 kcal/mol, hydrogen-bonded to the water dimer. A fifth motif is represented by Δ*G*_298.15_ of 0.56 and 0.85 kcal/mol, with a slight rotation of the water dimer. The last motif has Δ*G*_298.15_ values of 0.90 and 0.95 kcal/mol. In the 0.89 kcal/mol structure, the two water monomers connect three different hydroxyl groups with hydrogen bond distances of 1.75–1.81 Å and hydrogen-bond angles ranging from 157° to 162°.

**Figure 10:**
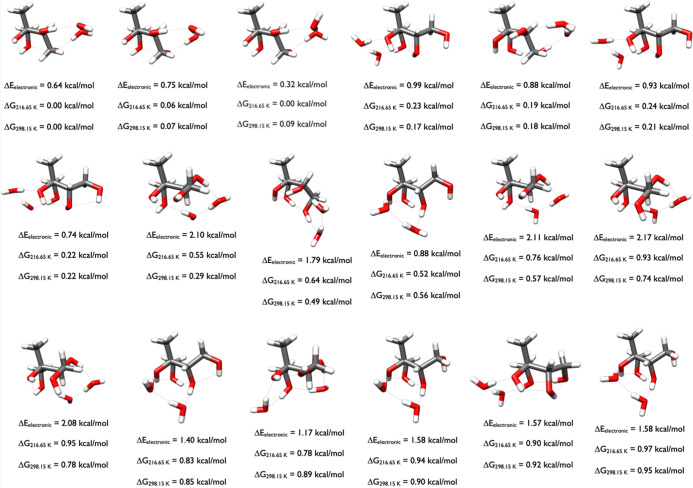
Lowest energy structures of 2-methylerythritol-(H_2_O)_2_ calculated at the DLPNO-CCSD­(T)/cc-pVnZ//ωB97X-D/6-31++G** (*n* = D, T, Q) level of theory, where the energies have been extrapolated to the CBS limit.

Figure 11 presents the 2-methylerythritol-(H_2_O)_3_ clusters. The structures displayed here are more varied than those for 2-methylthreitol (Figure 7). The five clusters with Gibbs free energy differences of 0.00, 0.62, 0.70, 0.91, and 0.97 kcal/mol form a pentameric structure with the three water molecules bridging two of the OH groups across 2-methylerythritol. The remaining structures show all the different ways that the varied positions of these waters comprise subtly different shapes. In this OGOLEM search of the PES, 3748 GFN2-xTB configurations were generated, which optimized to 2102 ωB97X-D geometries, and 753 of these were within 6 kcal/mol. The Δ*G*_298.15_ 0.34 kcal/mol structure was the 417th configuration on the ωB97X-D PES (Δ*E*_el_ of 5.02 and Δ*G*_298.15_ of 0.90 kcal/mol).

**Figure 11:**
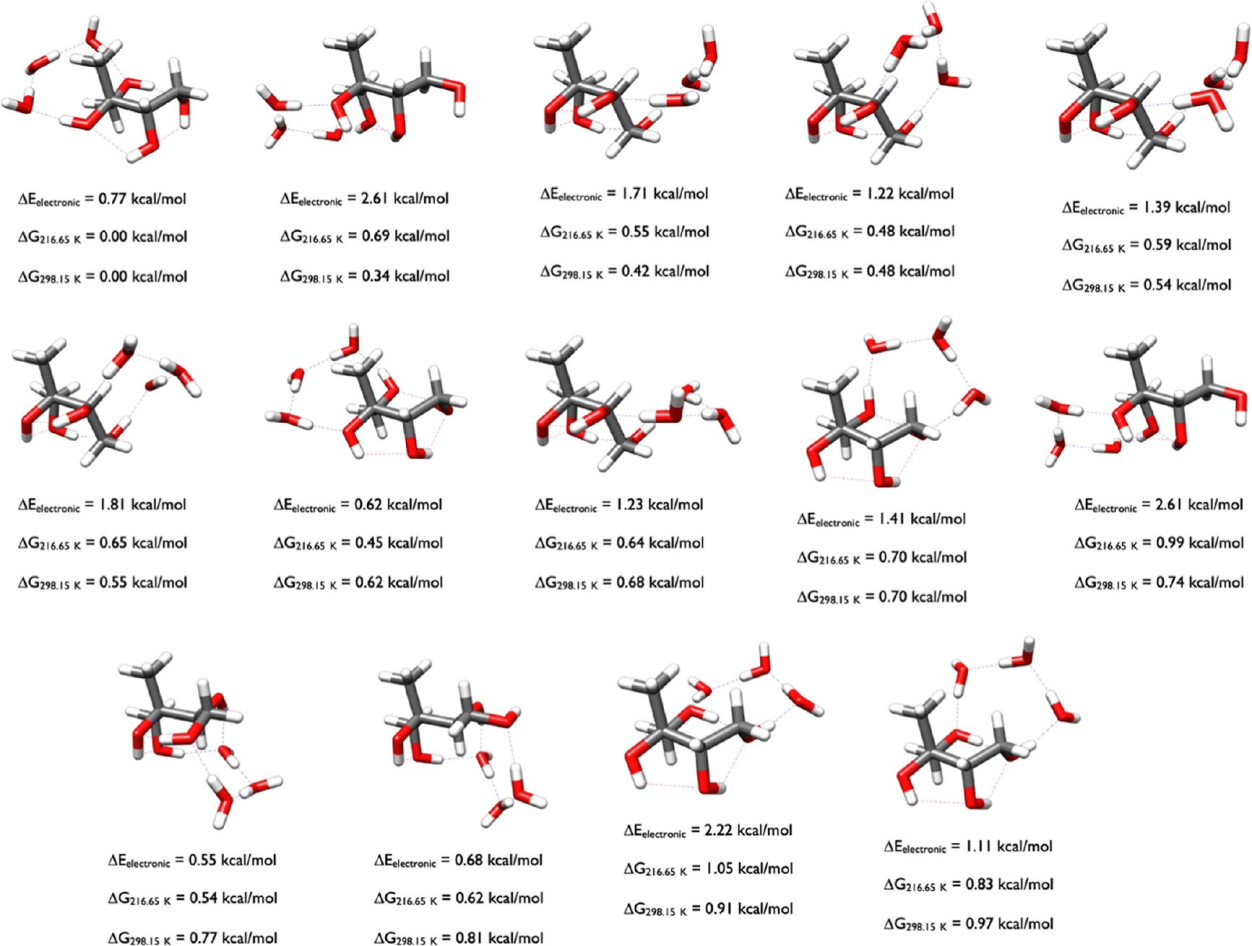
Lowest energy structures of 2-methylerythritol-(H_2_O)_3_ calculated at the DLPNO-CCSD­(T)/cc-pVnZ//ωB97X-D/6-31++G** (*n* = D, T, Q) level of theory, where the energies have been extrapolated to the CBS limit.

Figure 12 depicts the lowest-energy structures of 2-methylerythritol-(H_2_O)_4_. The OGOLEM search produced 3062 GFN2-xTB configurations which were geometry optimized with ωB97X-D, resulting in 1937 DFT structures. Of those, 365 were within 6 kcal/mol of the DFT minimum and DLPNO-CCSD­(T)/CBS calculations produced the energies in the figure. The minimum energy structure was the 339th ωB97X-D configuration (ωB97X-D Δ*E*_el_ of 5.90 and Δ*G*_298.15_ of 1.44 kcal/mol). This analysis reveals that even a 6 kcal/mol cutoff on the ωB97X-D Δ*E*_el_ values may not be sufficient to capture all of the low-lying free energy structures at ambient temperatures. The 2-methylerythritol-(H_2_O)_4_ structures have three waters forming a pentamer with two of the 2-methylerythritol OH groups, or four waters forming a hexamer-like structure with two of the 2-methylerythritol OH groups. The 2-methylerythritol moiety within these clusters is often identical, and the subtle differences in the water interactions with each other and the hydroxyl groups results in slightly different relative Gibbs free energies. The structures at 0.0, the second 0.41, and at 0.85 kcal/mol are congruent, as are those at 0.31 and 0.97 kcal/mol, the first 0.41 and 0.67 kcal/mol structures, the 0.87 and 0.94 kcal/mol structures, and the 0.53, 0.69, and 0.83 kcal/mol configurations. In the minimum free energy structure, the four waters form a hydrogen-bonded network that bridges two adjacent hydroxyl groups, which are held together by an intramolecular hydrogen bond. In the second minima at 0.29 kcal/mol, a water trimer network connects two hydroxyls on one side of the tetrol while the last water connects two OH groups on the other side. The four waters in the 0.31 kcal/mol configuration are arranged such that they form a tetramer with one hydroxyl and a pentamer with two other OH groups. In the first 0.41 kcal/mol structure, three waters form a pentamer with two OH groups and the fourth bridges the other two hydroxyls. Unlike the 2-methylthreitol-(H_2_O)_4_ structures in Figure 8, the water tetramer is not present, because different combinations of a three water network and a bridging monomer stabilize these structures. The diversity of structures corresponds to the diversity of the lowest energy conformations of the monomer displayed in Figure 4.

**Figure 12:**
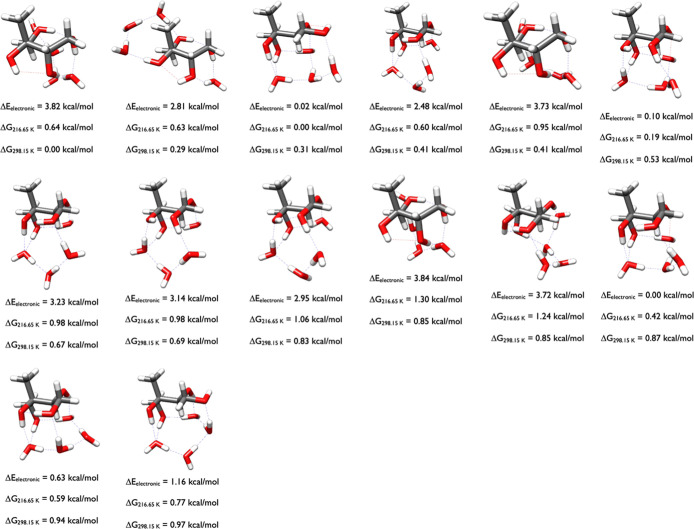
Lowest energy structures of 2-methylerythritol-(H_2_O)_4_ calculated at the DLPNO-CCSD­(T)/cc-pVnZ//ωB97X-D/6-31++G** (*n* = D, T, Q) level of theory, where the energies have been extrapolated to the CBS limit.

#### 2-Methyltetrols with Sulfuric Acid

Sulfuric acid has a unique ability to form multiple hydrogen bonds with bases and other molecules, and is one of the reasons it dominates in the formation of prenucleation clusters.^5,52–70^ As Figure 13 reveals, sulfuric acid has the flexibility to exist in the cis, trans or gauche conformations, thus maximizing its hydrogen bonds with 2-methylthreitol. The eight configurations within one kcal/mol of the Gibbs free energy minimum at 298 K are split between three trans, three cis, and two gauche conformations of sulfuric acid. Note how the high Δ*E*_el_ structures (2.31, 2.71, 4.12, and 2.77 kcal/mol) are all less than one kcal/mol from the minimum Δ*G*_298.15 K_ structure, and the minimum Gibbs free energy minimum itself has a high Δ*E*_el_ of 2.31 kcal/mol. This illustrates again that searching for these conformers is difficult, and the DLPNO-CCSD­(T) model chemistry has to be combined with the ωB97X-D thermodynamic corrections over a wide variety of structures.

**Figure 13:**
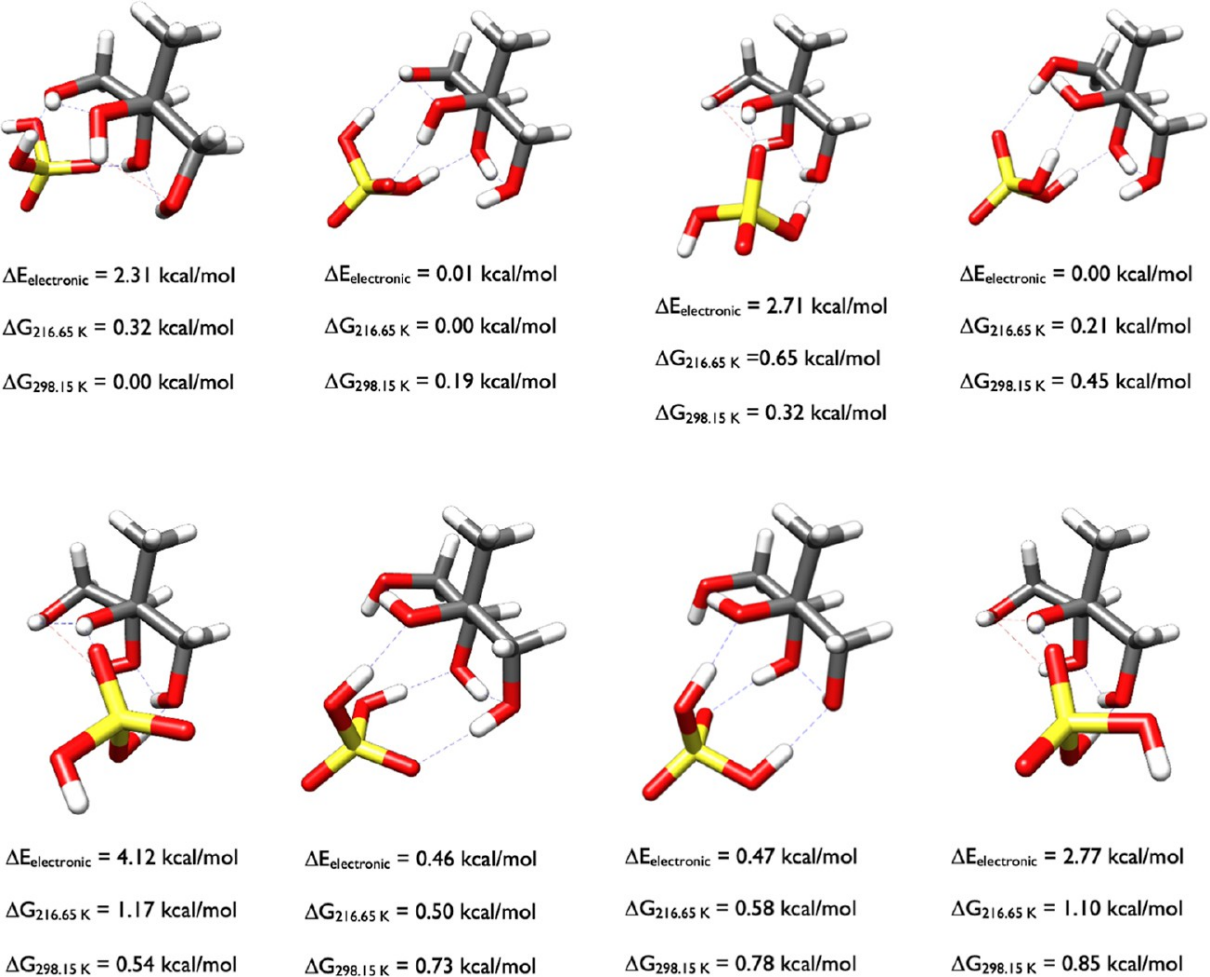
Lowest energy structures of 2-methylthreitol-H_2_SO_4_ calculated at the DLPNO-CCSD­(T)/cc-pVnZ//ωB97X-D/6-31++G** (*n* = D, T, Q) level of theory, where the energies have been extrapolated to the CBS limit.

Figure 14 shows the two 2-methylerythritol-H_2_SO_4_ clusters that are within one kcal/mol at 0.00 and 0.68 kcal/mol. Significantly, sulfuric acid is in the cis conformation in both of the clusters, and the overall complexes are almost congruent, with the only difference being that the front left hydroxyl group of 2-methylerythritol at 0.00 kcal/mol is pivoted left whereas the one at 0.68 kcal/mol is pointing up. These two structures are extremely stable relative to all other calculated configurations. In the OGOLEM simulation there were 934 GFN2-xTB minima, which were optimized to 468 ωB97X-D minima. DLPNO-CCSD­(T)/CBS single point calculations on the 156 ωB97X-D structures within 6 kcal/mol of the ωB97X-D minima resulted in only four structures within 2 kcal/mol of the DLPNO-CCSD­(T)/CBS Δ*G*_298.15_ minima, the two in the figure and two more at 1.88 and 1.92 kcal/mol.

**Figure 14:**
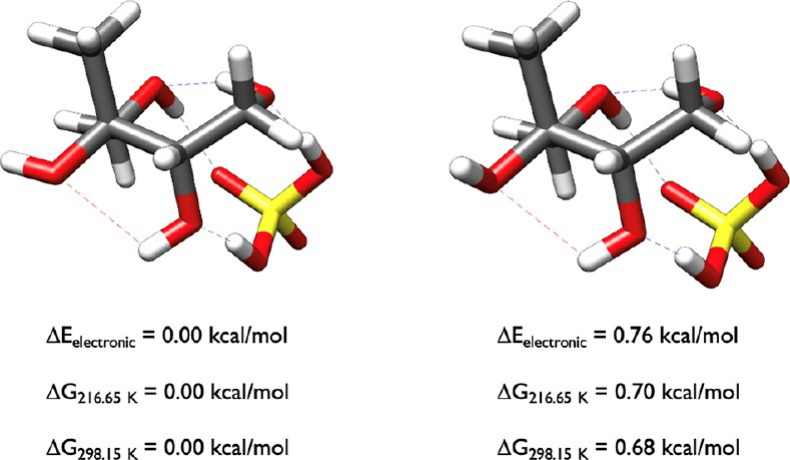
Lowest energy structures of 2-methylerythritol-H_2_SO_4_ calculated at the DLPNO-CCSD­(T)/cc-pVnZ//ωB97X-D/6-31++G** (*n* = D, T, Q) level of theory, where the energies have been extrapolated to the CBS limit.

#### 2-Methyltetrols with Water and Sulfuric Acid

The lowest Gibbs free energy structures of the 2-methylthreitol-H_2_SO_4_–H_2_O complex are shown in Figure 15. In every structure, sulfuric acid is present in the cis conformation and the water molecule stabilizes the sulfuric acid complex with 2-methylthreitol by donating a hydrogen bond to one of the oxygens on sulfuric acid and receiving a hydrogen bond from 2-methylthreitol in the six lowest Δ*G*_298.15_ structures. Note the only difference between the two isomers at 0.36 and 0.60 kcal/mol is a slight rotation of the water molecule, which changes the position of the dangling hydrogen.

**Figure 15:**
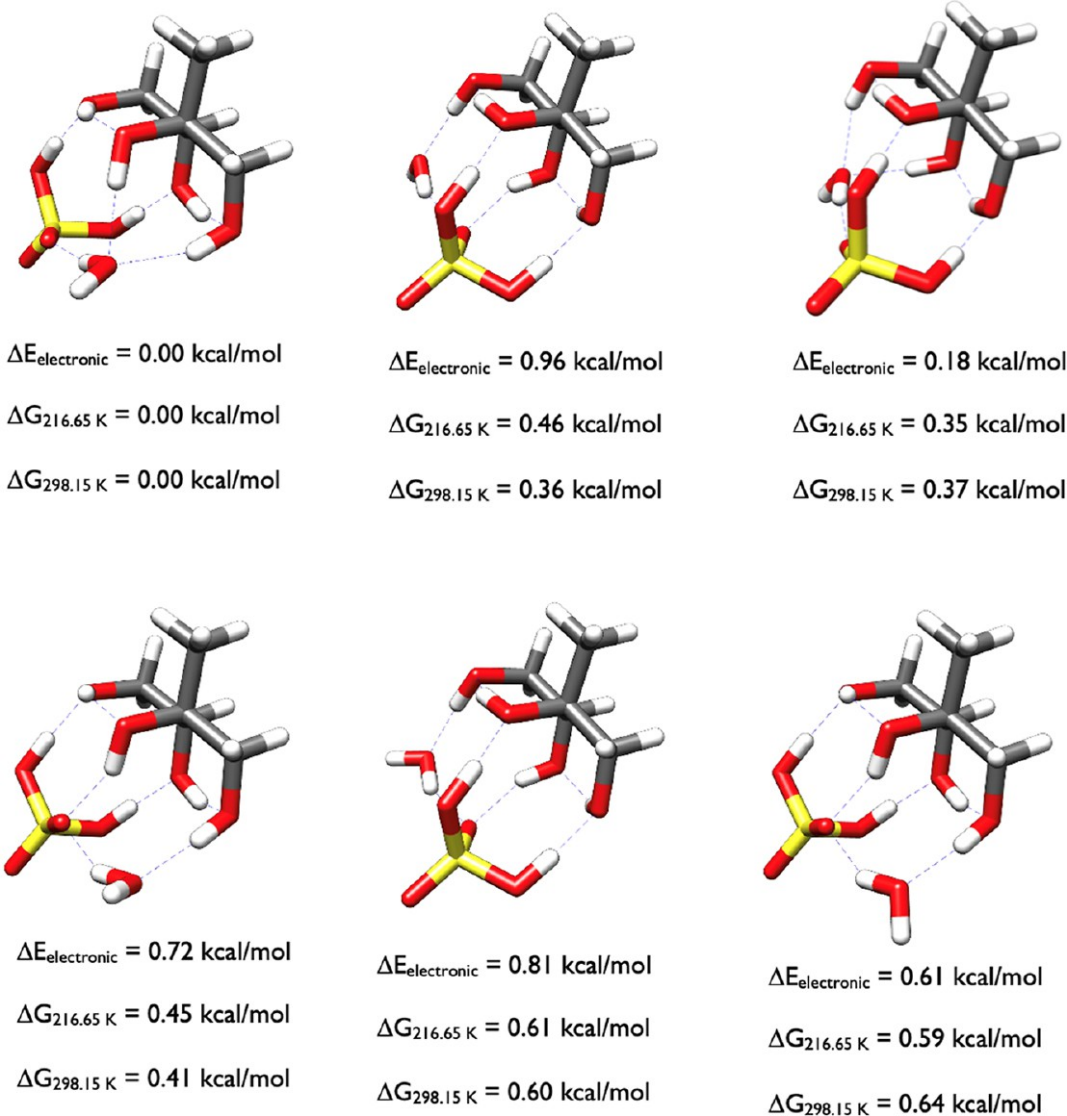
Lowest energy structures of 2-methylthreitol-H_2_SO_4_–H_2_O calculated at the DLPNO-CCSD­(T)/cc-pVnZ//ωB97X-D/6-31++G** (*n* = D, T, Q) level of theory, where the energies have been extrapolated to the CBS limit.

The two lowest Δ*G*_298.15_ structures for the 2-methylthreitol-H_2_SO_4_-(H_2_O)_2_ complex are displayed in Figure 16. The trans conformation of sulfuric acid bridges the waters and 2-methylthreitol in the minimum free energy structure. The water molecules bridge either side of the two larger molecules in the 1.0 kcal/mol free energy structure, and sulfuric acid’s conformation is gauche. The funnel method produced 1319 GFN2-xTB structures and 779 ωB97X-D structures, and 535 of these were subject to DLPNO-CCSD­(T) single point calculations. Of these 535, only 7 were within 2 kcal/mol of the DLPNO-CCSD­(T)/CBS free energy minimum. Besides the three in the figure, there were four more at 1.22, 1.38, 1.40, and 1.84 kcal/mol. The remaining 528 were higher than 2 kcal/mol.

**Figure 16:**
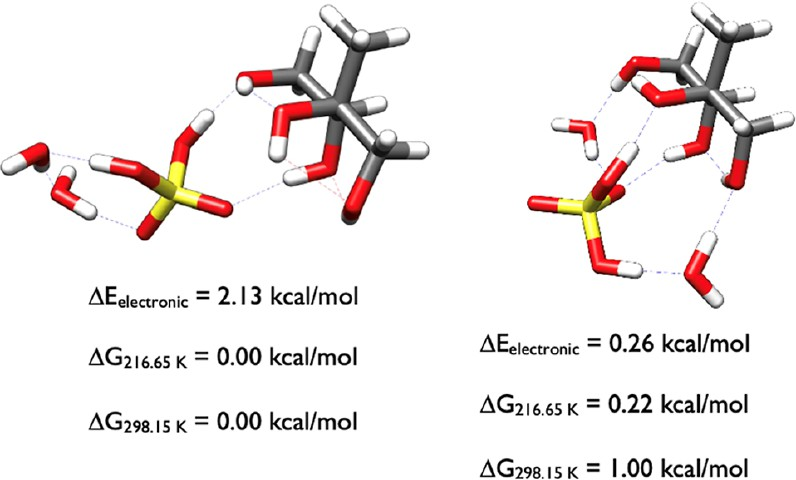
Lowest energy structures of 2-methylthreitol-H_2_SO_4_-(H_2_O)_2_ calculated at the DLPNO-CCSD­(T)/cc-pVnZ//ωB97X-D/6-31++G** (*n* = D, T, Q) level of theory, where the energies have been extrapolated to the CBS limit.

Adding a third water to the 2-methylthreitol-H_2_SO_4_ system results in 12 configurations within 1 kcal/mol of the minimum free energy structure (Figure 17). The structures with Δ*G*_298.15_ values of 0.00 and 0.69 kcal/mol are quite similar, with a water dimer and a monomer bridging the two larger molecules. The configurations at 0.04, 0.22, 0.29, 0.40, and 0.94 kcal/mol are also quite similar, with a water dimer on one side of sulfuric acid and the third water bridging H_2_SO_4_ and 2-methylthreitol. The structures at 0.35 and 0.90 kcal/mol have both the water dimer and the water monomer bridging the two larger molecules. Thus, a wide diversity of hydrogen bonding motifs lead to the diversity of structures in Figure 17. We note that from the funnel methodology, the final number of DFT structures within 6 kcal/mol was 232, and the minimum in the figure was #130, with the second structure (0.04 kcal/mol) being #154 in the ensemble.

**Figure 17:**
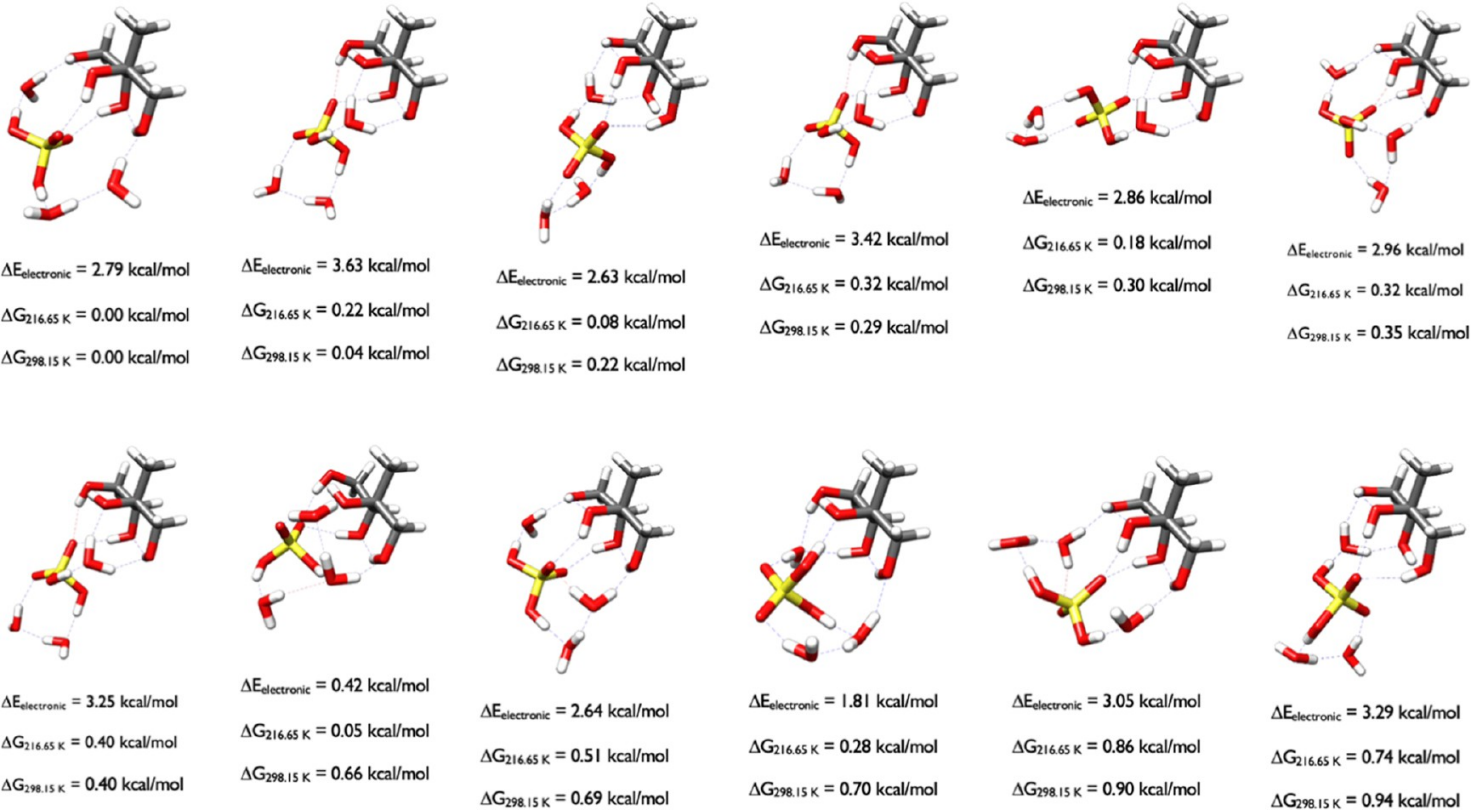
Lowest energy structures of 2-methylthreitol-H_2_SO_4_-(H_2_O)_3_ calculated at the DLPNO-CCSD­(T)/cc-pVnZ//ωB97X-D/6-31++G** (*n* = D, T, Q) level of theory, where the energies have been extrapolated to the CBS limit.

In sharp contrast to Figure 15, where only six 2-methylthreitol-H_2_SO_4_–H_2_O structures were within one kcal/mol of the Gibbs free energy minimum at 298 K, Figure 18 reveals that there are 29 2-methylerythritol-H_2_SO_4_–H_2_O isomers that are within this energy window. Here the trans conformation of sulfuric acid dominates, with it being present 20 times, whereas the cis conformation is only seen four times (0.23, 0.64 [first structure], 0.95 [second structure], and 1.0 [first structure] kcal/mol in Figure 18) and the gauche conformation five times (0.09, 0.67, 0.75, 0.94, and 0.97 kcal/mol). The greater diversity in structures results from the strong interaction between sulfuric acid and 2-methylerythritol, such that the water tends to be hydrogen bonded to sulfuric acid most of the time, and only bridges the two larger molecules five times (Δ*G*_298.15_ of 0.23, 0.64 [first structure], 0.95 [second structure], 0.97, 1.0 [first structure] kcal/mol in Figure 18). In the OGOLEM simulation 2626 GFN2 unique structures were generated, and ωB97X-D geometry optimization reduced this to 1158 DFT structures, of which 332 were within 6 kcal/mol of the DFT minima. The 0.95 kcal/mol structure in the figure is the 332nd configuration (Δ*E*_el_ 5.98 and Δ*G*_298.15_ 2.26 kcal/mol), revealing again that despite an extensive semiempirical search routine and a relatively high cutoff on DFT electronic energies, it is always possible to miss low-lying DLPNO-CCSD­(T) free energy structures.

**Figure 18:**
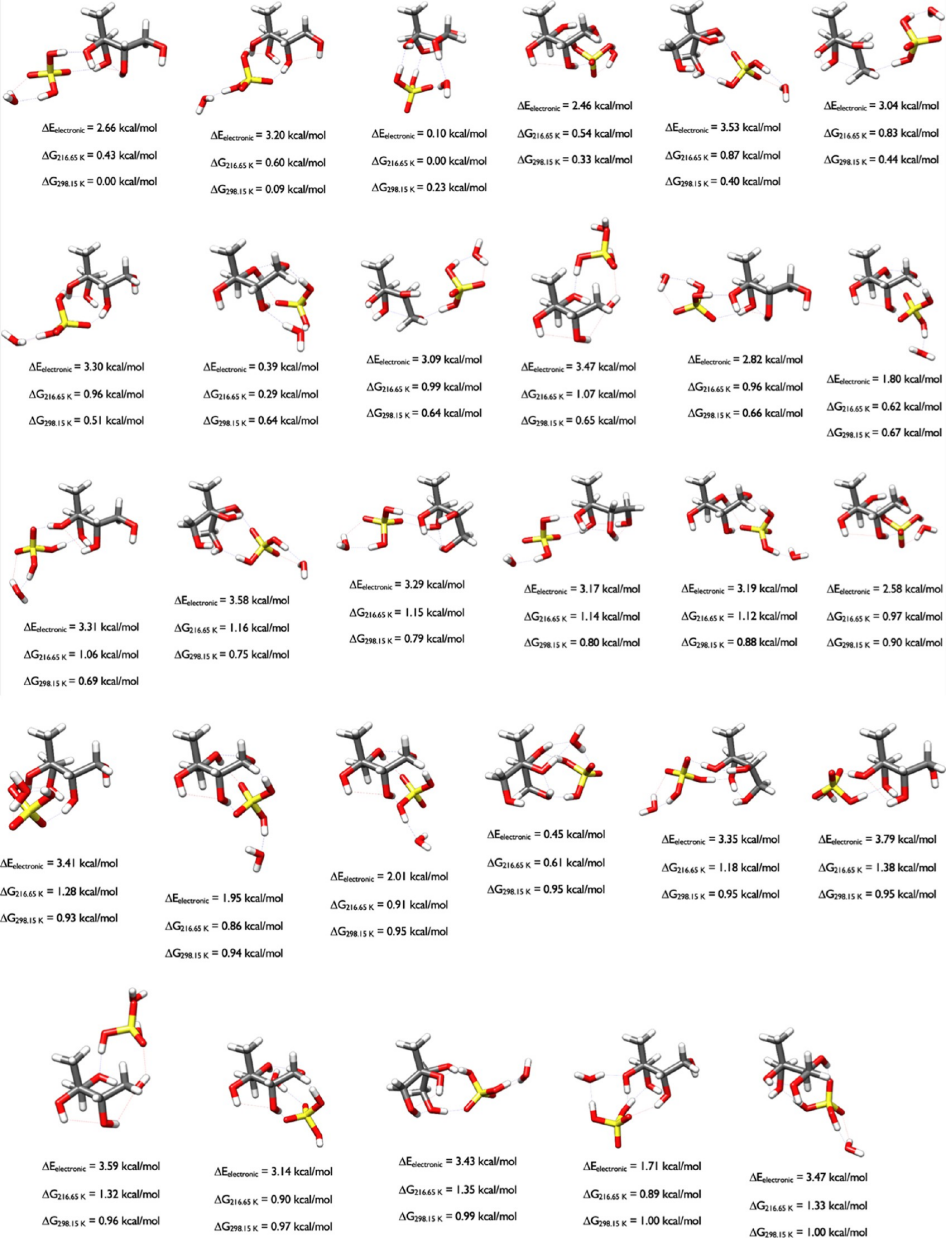
Lowest energy structures of 2-methylerythritol-H_2_SO_4_–H_2_O calculated at the DLPNO-CCSD­(T)/cc-pVnZ//ωB97X-D/6-31++G** (*n* = D, T, Q) level of theory, where the energies have been extrapolated to the CBS limit.

The three 2-methylerythritol-H_2_SO_4_ lowest free energy structures in Figure 19 are nearly identical, with the only differences being the interactions with the two waters in these complexes. The water nearest the sulfuric acid stabilizes it in a gauche conformation while the water closest to 2-methylerythritol stabilizes several OH groups.

**Figure 19:**
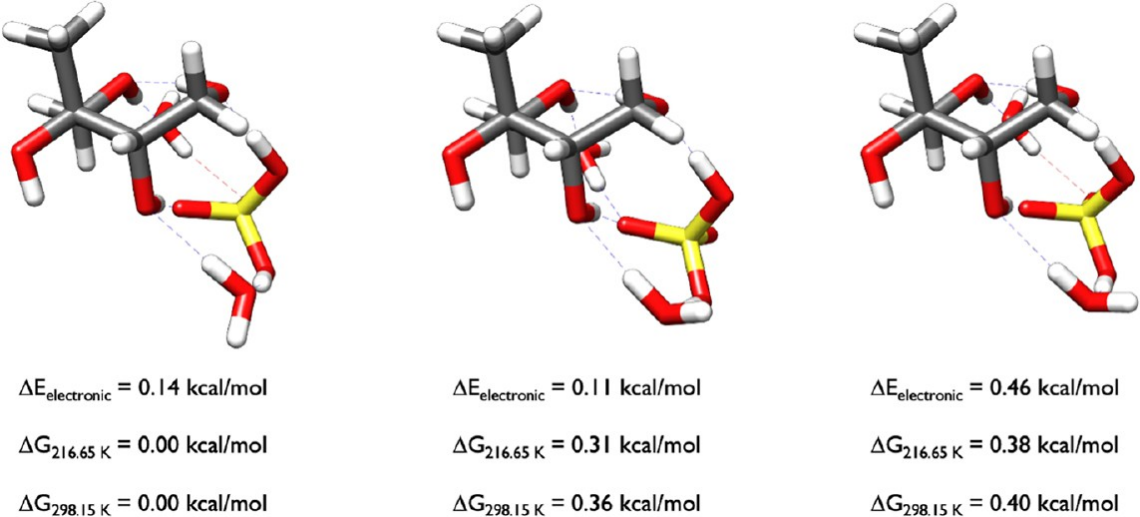
Lowest energy structures of 2-methylerythritol-H_2_SO_4_-(H_2_O)_2_ calculated at the DLPNO-CCSD­(T)/cc-pVnZ//ωB97X-D/6-31++G** (*n* = D, T, Q) level of theory, where the energies have been extrapolated to the CBS limit.

Figure 20 indicates the lowest free energy clusters of 2-methylerythritol-H_2_SO_4_-(H_2_O)_3_. The conformations at 0.00 and 0.10 kcal/mol are almost completely congruent, with the only difference being that the water monomers that are hydrogen bonded to 2-methylerythritol are orientated differently, horizontally (0.00 kcal/mol) and vertically (0.10 kcal/mol). Similarly, for those at 0.14 and 0.32 kcal/mol, the structures are identical with the exception of the water monomer rotation. The configuration at 0.55 kcal/mol differs in that now the water dimer bridges the two larger molecules while the water monomer stabilizes sulfuric acid.

**Figure 20:**
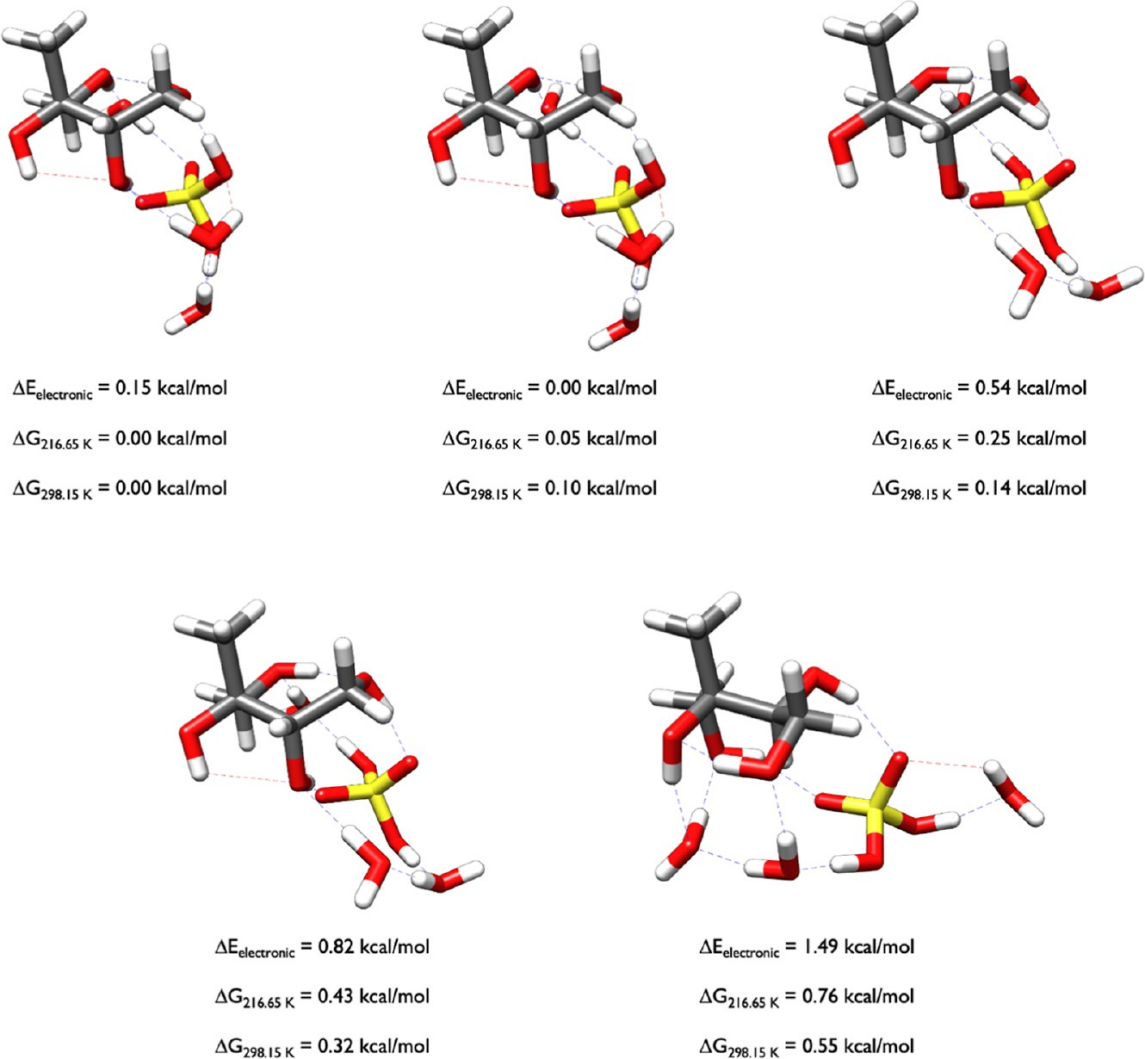
Lowest energy structures of 2-methylerythritol-H_2_SO_4_-(H_2_O)_3_ calculated at the DLPNO-CCSD­(T)/cc-pVnZ//ωB97X-D/6-31++G** (*n* = D, T, Q) level of theory, where the energies have been extrapolated to the CBS limit.

### Gibbs Free Energies for the Formation of Clusters

Table 1 displays the overall Gibbs free energies for the stepwise formation of the most stable 2-methylthreitol isomer with one to four waters at temperatures stemming from the top (217 K) to the bottom (298 K) of the troposphere. This represents gas-phase reactions, where two and three body collisions are responsible for the sequential buildup of larger complexes from smaller ones. The stepwise Δ*G*° values were computed using the lowest-energy structures from each figure with multiconformer corrections applied. For reference, the corresponding Δ*G*° values computed using only the global minimum structures, without multiconformer contributions, are listed in parentheses.

**Table 1:** Sequential DLPNO-CCSD­(T)/CBS//ωB97X-D/6-31++G** Gibbs Free Energies (kcal/mol) for the Step-Wise Addition of Water to 2-Methylthreitol^a^

reaction	Δ*G* (216.65 K)	Δ*G* (273.15 K)	Δ*G* (298.15 K)
2-methylthreitol + H_2_O → 2-methylthreitol-H_2_O	–1.48 (−1.00)	0.35 (0.84)	1.15 (1.65)
2-methylthreitol-H_2_O → 2-methylthreitol-(H_2_O)_2_	–2.13 (−2.12)	–0.35 (−0.30)	0.45 (0.50)
2-methylthreitol-(H_2_O)_2_ → 2-methylthreitol-(H_2_O)_3_	–0.92 (−0.57)	1.03 (1.19)	1.85 (1.97)
2-methylthreitol-(H_2_O)_3_ → 2-methylthreitol-(H_2_O)_4_	–1.16 (−0.97)	0.71 (1.04)	1.56 (1.93)

a Values include the multiconformer contribution, while values in parentheses were computed using global minimum structures only.

As would be expected, the lower the temperature the lower the entropic effect of cluster formation, so that the overall free energies are negative at 217 K and become positive as temperature increases. The sequential free energies, derived from the overall free energies, generally become more positive as clusters grow bigger, but as some clusters are particularly stable this is only a general trend.

Table 2 shows similar trends in the Gibbs free energies at 216.65 and 298.15 K as with Table 1, with the actual numerical differences reflecting the differing stabilities of the lowest Gibbs free energy structures for the different diastereoisomers complexed with one to four waters.

**Table 2:** Sequential DLPNO-CCSD­(T)/CBS//ωB97X-D/6-31++G** Gibbs Free Energies (kcal/mol) for the Step-Wise Addition of Water to 2-Methylerythritol^a^

reaction	Δ*G* (216.65 K)	Δ*G* (273.15 K)	Δ*G* (298.15 K)
2-methylerythritol + H_2_O → 2-methylerythritol-H_2_O	–1.37 (−1.35)	0.44 (0.43)	1.24 (1.21)
2-methylerythritol-H_2_O → 2-methylerythritol-(H_2_O)_2_	–2.09 (−1.55)	–0.28 (0.20)	1.16 (0.97)
2-methylerythritol-(H_2_O)_2_ → 2-methylerythritol-(H_2_O_)3_	–1.65 (−1.90)	0.17 (−0.17)	0.45 (0.59)
2-methylerythritol(H_2_O)_3_ → 2-methylerythritol-(H_2_O)_4_	–0.51 (−0.62)	0.95 (1.11)	1.75 (1.88)

a Values include the multiconformer contribution, while values in parentheses were computed using global minimum structures only.

Adding sulfuric acid to the mix results in more negative energies, a consequence of the greater hydrogen bonding ability of sulfuric acid relative to everything else in the atmosphere. Tables 3 and 4 contain the Gibbs free energies for reactions of H_2_SO_4_, each diastereoisomer, and 0 – 3 water molecules. Two interesting trends are present in the data. First, formation of the 2-methylerythritol-H_2_SO_4_ complex is favored over the same complex with 2-methylthreitol. This preferential binding of H_2_SO_4_ to 2-methylerythritol could be due to its 3 intramolecular hydrogen bonds, compared to the 4 intramolecular hydrogen bonds in 2-methylthreitol. Prior studies have shown that such intramolecular stabilization can reduce the clustering free energy via an energetic penalty of breaking the intramolecular hydrogen bonds before intermolecular hydrogen bonds are formed.^71–73^ Second, the sequential energies for adding water are more negative for the 2-methylthreitol system. The consequences of these changes will be discussed when we estimate the concentrations of all species in the next section.

**Table 3:** Sequential DLPNO-CCSD­(T)/CBS//ωB97X-D/6-31++G** Gibbs Free Energies (kcal/mol) for Reactions of 2-Methylthreitol, H_2_SO_4_ (SA), and H_2_O (W)^a^

reaction	Δ*G* (216.65 K)	Δ*G* (273.15 K)	Δ*G* (298.15 K)
2-methylthreitol + SA → 2-methylthreitol-SA	–8.07 (−7.63)	–6.03 (−5.50)	–5.01 (−4.57)
2-methylthreitol-SA + W → 2-methylthreitol-SA-W	–5.18 (−5.28)	–2.84 (−3.07)	–1.92 (−2.08)
2-methylthreitol-SA-W + W → 2-methylthreitol-SA-W2	–1.36 (−1.18)	0.26 (0.07)	0.95 (0.62)
2-methylthreitol-SA-W_2_ + W → 2-methylthreitol-SA-W_3_	–3.12 (−2.49)	–1.09 (−0.26)	–0.17 (0.73)

a Values include the multiconformer contribution, while values in parentheses were computed using global minimum structures only.

**Table 4:** Sequential DLPNO-CCSD­(T)/CBS//ωB97X-D/6-31++G** Gibbs Free Energies (kcal/mol) for Reactions of 2-Methylerythritol, H_2_SO_4_ (SA), and H_2_O (W)^a^

reaction	Δ*G* (216.65 K)	Δ*G* (273.15 K)	Δ*G* (298.15 K)
2-methylerythritol + SA → 2-methylerythritol-SA	–8.41 (−9.21)	–5.95 (−6.81)	–4.86 (−5.75)
2-methylerythritol-SA + W → 2-methylerythritol-SA-W	–3.03 (−1.77)	–1.77 (−0.38)	–1.04 (0.23)
2-methylerythritol-SA-W + W → 2-methylerythritol-SA-W2	–1.48 (−2.38)	1.04 (−0.04)	1.98 (1.10)
2-methylerythritol-SA-W_2_ + W → 2-methylerythritol-SA-W_3_	4.91 (5.07)	8.70 (8.89)	10.39 (10.59)

a Values include the multiconformer contribution, while values in parentheses were computed using global minimum structures only.

Overall, sulfuric acid clusters are more negative than nonsulfuric acid ones because sulfuric acid has multiple hydrogen bonding opportunities as a consequence of its unique structure in all of its conformations (cis, trans, and gauche). Moreover, sulfuric acid is a strong acid, so high acidity increases stability of the clusters in effective aerosol formation.^45,46^

Finally, Table 4 highlights the significant effect that contributions from higher-energy conformers can have on the calculated free energies. For example, the 2-methylerythritol-SA-W_0–3_ Δ*G*° values change by upward of 1 kcal/mol when multiconformer contributions are included. The Δ*G*° of 2-methylerythritol-SA-W changes by over 1 kcal/mol at all three temperatures. This complex has 29 structures within 1 kcal/mol of the global minimum and a multiconformer contribution (considering all structures within 6 kcal/mol of the minimum) of −1.6 to −1.8 kcal/mol, depending on the temperature. In contrast, the Δ*G*° for other complexes are not as largely affected by the contribution of other conformers.

### Estimated Concentrations of Clusters in the Atmosphere

The Gibbs free energies at 298.15 K were used to determine estimates of the equilibrium concentrations of the clusters at the bottom of the troposphere. This was done by first calculating the equilibrium constant K for each cluster formation reaction since, *K* = e^–Δ*G*°/*RT*^. These equilibrium constants are equivalent to the equilibrium concentrations of the cluster being formed over those of the monomers raised to the necessary powers. Additionally, mass balance equations were added to ensure that no molecules were gained or lost in the simulation. These concentration and mass balance equations were solved simultaneously for the final equilibrium concentrations, provided initial guesses for the total concentration of each monomer are included. We assumed a closed system of 2-methylthreitol, 2-methylerythritol, sulfuric acid, and three waters, and used the equilibrium constants for every possible reaction between each of these species. Most of the Δ*G*° values are in the tables, and we used previously calculated values for formation of the H_2_SO_4_–H_2_O_*n*_ clusters (−2.01 for one water, −3.14 for two waters, and −3.42 kcal/mol for three waters at 298 K).^44^ For these simulations, we used a water concentration of 7.7 × 10^17^ cm^–3^ at 298 K, which corresponds to 100% humidity at the bottom of the troposphere.^74^ We used a sulfuric acid concentration of 5 × 10^7^ cm^–3^, which is atmospherically relevant.^74^ For the tetrols we used a concentration of 5.18 × 10^12^ cm^–3^, based on the experimental measurement of 2,3,4-pentanetriol at 300 K as explained by Claeys et al.^12^ This was measured in aerosol-phase samples, reflecting condensed-phase tetrols rather than gas-phase abundances. Therefore, we also used values of 5 × 10^7^ cm^–3^ and 5 × 10^4^ cm^–3^ in order to aid in our analysis. The results of these calculations can be seen in Table 5.

**Table 5:** Equilibrium Concentrations of Clusters that Form at More than 1 cm^–3^ at 298 K^a^

cluster	[tetrol] 5.18 × 10^12^ cm^–3^	[tetrol] 5 × 10^7^ cm^–3^	[tetrol] 5 × 10^4^ cm^–3^
SA	2.3 × 10^7^	2.3 × 10^7^	2.3 × 10^7^
2-methylerythritol	5.2 × 10^12^	5.0 × 10^7^	5.0 × 10^4^
2-methylthreitol	5.2 × 10^12^	5.0 × 10^7^	5.0 × 10^4^
W	7.7 × 10^17^	7.7 × 10^17^	7.7 × 10^17^
SA-W_1_	2.2 × 10^7^	2.2 × 10^7^	2.2 × 10^7^
SA-W_2_	4.6 × 10^6^	4.6 × 10^6^	4.6 × 10^6^
SA-W_3_	2.3 × 10^5^	2.3 × 10^5^	2.3 × 10^5^
2-methylerythritol-W_1_	2.0 × 10^10^ (2.1 × 10^10^)	1.9 × 10^5^ (2.0 × 10^5^)	1.9 × 10^2^ (2.0 × 10^2^)
2-methylerythritol-W_2_	8.8 × 10^7^ (1.3 × 10^8^)	8.5 × 10^2^ (1.2 × 10^3^)	<1 (1.2)
2-methylerythritol-W_3_	1.3 × 10^6^ (1.5 × 10^6^)	12 (14)	<1
2-methylthreitol-W_1_	2.3 × 10^10^ (1.0 × 10^10^)	2.2 × 10^5^ (9.6 × 10^4^)	2.2 × 10^2^ (96)
2-methylthreitol-W_2_	3.4 × 10^8^ (1.3 × 10^8^)	3.3 × 10^3^ (1.3 × 10^3^)	3.3 (1.3)
2-methylthreitol-W_3_	4.7 × 10^5^ (1.5 × 10^5^)	4.5 (1.5)	≪1
SA-2-methylerythritol	1.8 × 10^4^ (8.0 × 10^4^)	<1	≪1
SA-2-methylerythritol-W	3.2 × 10^3^ (1.7 × 10^3^)	<1	≪1
SA-2-methylerythritol-W_2_	3.6 (9.8)	≪1	≪1
SA-2-methylerythritol-W_3_	≪ 1	≪1	≪1
SA-2-methylthreitol	2.3 × 10^4^ (1.1 × 10^4^)	<1	≪1
SA-2-methylthreitol-W	1.8 × 10^4^ (1.2 × 10^4^)	<1	≪1
SA-2-methylthreitol-W_2_	1.2 × 10^2^ (1.3 × 10^2^)	≪1	≪1
SA-2-methylthreitol-W_3_	4.8 (1.2)	≪1	≪1

a Initial concentrations of monomers are 5 × 10^7^ cm^–3^ for sulfuric acid and 7.7 × 10^17^ cm^–3^ for water. We used three values for the tetrols, an upper limit of 5.18 × 10^12^ cm^–3^, the same concentration as sulfuric acid (5 × 10^7^ cm^–3^), and 5 × 10^4^ cm^–3^. Values in parentheses are those computed with free energies of global minimum structures. Those not in parentheses are computed using free energies that include the multiconformer contribution. If only one value is listed, the results do not change when including the multiconformer contribution. SA = sulfuric acid. W = water.

In Table 5, values in parentheses are those computed with free energies of global minimum structures, while those not in parentheses account for the multiconformer contribution. If only one value is reported, including multiple conformers does not change the result. Complexes containing both 2-methylerythritol and SA show notable differences in concentration when considering multiple conformers. When the initial tetrol concentration is 5.18 × 10^12^ cm^–3^, the SA-2-methylerythritol concentration exhibits the largest change in the table—a 4.44× decrease when including multiple conformers. As previously mentioned, these 2-methylerythritol-SA containing complexes also incur relatively large changes in Gibbs free energies of formation (∼1 kcal/mol) compared to other complexes in the study, as shown in Table 4. While these changes in concentration can be large, none vary by a full order of magnitude, whereas concentrations between different complexes often differ by at least that much. Therefore, our qualitative comparison of complex concentrations is unaffected by the inclusion of multiconformer contributions to the free energy.

At the upper limit of the tetrol concentration, 5.18 × 10^12^ cm^–3^, we observe that even though the Δ*G*° values in the tables are more negative whenever sulfuric acid is involved in a cluster, the tetrols themselves are predicted to form significant amounts of clusters with water. The concentrations of each tetrol with one or two waters exceeds the predicted concentrations of sulfuric acid with one or two waters. This suggests that tetrols exhibit notable hygroscopic behavior, likely due to their multiple hydroxyl functional groups enabling strong hydrogen bonding with water. Once the third water is added, the concentrations of the tetrol-W_3_ and SA-W_3_ clusters are quite similar, despite the much lower concentration of sulfuric acid in the simulation. Formation of the sulfuric acid–tetrol–water complexes are less than those for sulfuric acid and water, or either tetrol and water, leading us to surmise that it is unlikely that sulfuric acid and the tetrols will grow prenucleation clusters to a size that would lead to NPF. When we reduce the concentration of the tetrols to the same value as for sulfuric acid, as shown in the middle column of Table 5, we find that now sulfuric acid dominates the formation of all clusters. The tetrol-water concentrations are now two to 4 orders of magnitude lower than the sulfuric acid–water concentrations, revealing that despite having four hydroxyl groups the interactions between sulfuric acid and water are stronger. The tetrol-sulfuric acid–water clusters are much lower than at the higher tetrol concentrations. With even lower concentrations of tetrols (5 × 10^4^ cm^–3^), trends are similar.

In Table 5, the initial concentrations of SA and the tetrols were not held constant. We have recalculated the concentrations using fixed concentrations of SA and the tetrols, and reported the results in the Supporting Information. Still, the SA-tetrol-water concentrations are much lower than those of SA-water and either tetrol-water.

These results lead to the conclusion that the tetrols formed from photooxidation of isoprene are unlikely to participate in the formation of prenucleation complexes. In contrast, there have been numerous studies of the ability of sulfuric acid, waters, other acids, and various bases to form prenucleation complexes.^5–7,9,41,44–46,52–63,65–70^ ^,^ ^75–103^ We also know that highly oxygenated molecules are present in larger aerosols, but the details of when in aerosol formation highly oxygenated molecules are incorporated is still an area of active research.^7,16,67,101^

## Conclusions

The diastereomeric tetrols, 2-methylthreitol and 2-methylerythritol, are produced from the photooxidation of isoprene, and contain four hydroxyl groups. We have completed a comprehensive conformational search of both tetrols, as well as an extensive exploration of the PESs of these tetrols complexed with sulfuric acid and water. We have reported the vast array of structures that are within 1 kcal/mol of the DLPNO-CCSD­(T)/CBS//ωB97X-D/6-31++G** minimum for each system. We have used these high level Δ*G*° values for each system to estimate the concentrations of all the possible complexes in the lower troposphere. At the upper limit of tetrol concentrations, we find that the two diastereomers will bind to one to three water molecules in high concentrations. However, formation of sulfuric acid–tetrol–water complexes lead to lower concentrations, suggesting that these tetrols are unlikely to be involved in the formation of prenucleation clusters that will lead to further aerosol growth.

This study adds to the body of evidence that certain atmospherically relevant organics, such as tetrols, do not significantly enhance cluster formation with sulfuric acid. To make further progress, efforts might focus on identifying functional groups necessary for a stable cluster through the “clustering of functional groups” approach, rather than relying solely on identified atmospheric species.^3^ Additionally, the integration of machine learning in chemical modeling provides the ability to evaluate energies of conformers more quickly, enabling studies of large accretion products (e.g., covalently bound organic dimers) that were previously computationally inaccessible due to their large size and high torsional complexity. The search for organic molecules that lead to prenucleation continues.

## Supplementary Material



## Data Availability

All of the coordinates for the structures reported in this paper, as well as two spreadsheets that contains all of the energetic results, can be found here: https://github.com/olongsworth/shields-group.

## Notes

Multiconformer contributions to the free energy for each complex, and a table of concentrations with fixed initial concentrations of the tetrols and sulfuric acid (PDF)
