# Supplementary material for: Computational Exploration of the Ability of the 2‑Methyltetrols Produced from Photooxidation of Isoprene to Form Prenucleation Complexes
Source: ACS Omega. 2025 Jun 6;10(23):24811–31. doi: 10.1021/acsomega.5c01981 (PMC12177631; doi:10.1021/acsomega.5c01981)
Supplement: Supplementary file 1 [file ao5c01981_si_001.pdf]

Supplementary Information for Computational Exploration of the Ability of the 2-Methyltetrols produced from Photooxidation of Isoprene to form Prenucleation Complexes

Conor J. Bready, Alexandra E. Sorescu, Caroline S. Glick and George C. Shields*

Furman University, Department of Chemistry, 3300 Poinsett Highway, Greenville, SC 29613, USA

email george.shields@furman.edu

Table S1. Stabilization of minimum Gibbs free energy due to multiple conformers. SA = Sulfuric Acid. W = Water.

Cluster	ΔG (216.65 K)	ΔG (273.15 K)	ΔG (298.15 K)
2-methylthreitol	-0.42	-0.46	-0.49
2-methylthreitol-W	-0.90	-0.95	-0.98
2-methylthreitol-W_2	-0.92	-1.00	-1.03
2-methylthreitol-W_3	-1.26	-1.17	-1.16
2-methylthreitol-W_4	-1.46	-1.50	-1.53
2-methylthreitol-SA	-0.86	-0.99	-0.93
2-methylthreitol-SA-W	-0.76	-0.77	-0.77
2-methylthreitol-SA-W_2	-0.94	-0.57	-0.44
2-methylthreitol-SA-W_3	-1.57	-1.40	-1.34
2-methylthreitol-SA-W_4	-0.16	-0.13	-0.12
2-methylerythritol	-1.16	-1.26	-1.30
2-methylerythritol-W	-1.18	-1.24	-1.28
2-methylerythritol-W_2	-1.73	-1.72	-1.73
2-methylerythritol-W_3	-1.48	-1.38	-1.23
2-methylerythritol-W_4	-1.36	-1.54	-1.36
2-methylerythritol-SA	-0.35	-0.39	-0.41
2-methylerythritol-SA-W	-1.62	-1.78	-1.68
2-methylerythritol-SA-W_2	-0.72	-0.71	-0.70
2-methylerythritol-SA-W_3	-0.88	-0.90	-0.91

Table S2. Overall DLPNO-CCSD(T)/CBS//ωB97X-D/6-31++G** Gibbs free energies (kcal/mol) for reactions of 2-methylthreitol and H_2O to make the indicated product clusters. Values include the multiconformer contribution, while values in parentheses were computed using global minimum structures only.

Reaction	ΔG (216.65 K)	ΔG (273.15 K)	ΔG (298.15 K)
2-methylthreitol + H_2O → 2-methylthreitol-H_2O	-1.48 (-1.00)	0.35 (0.84)	1.15 (1.65)
2-methylthreitol + 2 H_2O → 2-methylthreitol-$(H_2O)_2$	-3.61 (-3.11)	0 (0.53)	1.61 (2.14)
2-methylthreitol + 3 H_2O → 2-methylthreitol-$(H_2O)_3$	-4.53 (-3.69)	1.02 (1.73)	3.45 (4.12)
2-methylthreitol + 4 H_2O → 2-methylthreitol-$(H_2O)_4$	-5.69 (-4.65)	1.73 (2.77)	5.01 (6.05)

Table S3. Equilibrium concentrations of clusters that form at more than 1 cm^{-3} at 298 K. Initial concentrations of monomers are 5 x 10^7 cm^{-3} for sulfuric acid and 7.7 x 10^{17} cm^{-3} for water. We used three values for the tetrols, an upper limit of 5.18 x 10^{12} cm^{-3} ,the same concentration as sulfuric acid (5 x 10^7 cm^{-3}), and 5 x 10^4 cm^{-3}. Initial concentrations were held constant. Values in parentheses are those computed with free energies of global minimum structures. Those not in parenthesis are computed using free energies that include the multiconformer contribution. If only one value is listed, these the results do not change when including the multiconformer contribution. SA = Sulfuric Acid. W = Water.

Cluster	[Tetrol] 5.18 x 10^{12} cm^{-3}	[Tetrol] 5 x 10^7 cm^{-3}	[Tetrol] 5 x 10^4 cm^{-3}
SA	5.0 x 10^7	5.0 x 10^7	5.0 x 10^7
2-methylerythritol	5.2 x 10^{12}	5.0 x 10^7	5.0 x 10^4
2-methylthreitol	5.2 x 10^{12}	5.0 x 10^7	5.0 x 10^4
W	7.7 x 10^{17}	7.7 x 10^{17}	7.7 x 10^{17}
SA-W_1	4.7 x 10^7	4.7 x 10^7	4.7 x 10^7
SA-W_2	9.8 x 10^6	9.8 x 10^6	9.8 x 10^6
SA-W_3	4.9 x 10^5	4.9 x 10^5	4.9 x 10^5
2-methylerythritol - W_1	2.0 x 10^{10} (2.1 x 10^{10})	1.9 x 10^5 (2.0 x 10^5)	1.9 x 10^2 (2.0 x 10^2)
2-methylerythritol – W_2	8.8 x 10^7 (1.3 x 10^8)	8.5 x 10^2 (1.2 x 10^3)	<1 (1.2)
2-methylerythritol – W_3	1.3 x 10^6 (1.5 x 10^6)	12.5 (14.3)	<1
2-methylthreitol – W_1	2.3 x 10^{10} (1.0 x 10^{10})	2.3 x 10^5 (9.7 x 10^4)	2.3 x 10^2 (97)
2-methylthreitol – W_2	3.4 x 10^8 (1.4 x 10^8)	3.3 x 10^3 (1.3 x 10^3)	3.3 (1.3)
2-methylthreitol – W_3	4.7 x 10^5 (1.5 x 10^5)	4.5 (1.5)	<<1
SA – 2-methylerythritol	3.8 x 10^4 (1.7 x 10^5)	<1 (1.7)	<<1
SA – 2-methylerythritol - W	7.0 x 10^3 (3.7 x 10^3)	<1	<<1
SA – 2-methylerythritol – W_2	7.7 (20.8)	<<1	<<1
SA – 2-methylerythritol – W_3	<< 1 (<<1)	<<1	<<1
SA - 2-methylthreitol	5.0 x 10^4 (2.4 x 10^4)	<1	<<1
SA – 2-methylthreitol - W	4.0 x 10^4 (2.5 x 10^4)	<1	<<1

SA – 2-methylthreitol – W_2	2.5×10^2 (2.7×10^2)	<<1	<<1
SA – 2-methylthreitol – W_3	10.4 (2.5)	<<1	<<1
